# Levinson’s type generalization of the Jensen inequality and its converse for real Stieltjes measure

**DOI:** 10.1186/s13660-016-1274-y

**Published:** 2017-01-03

**Authors:** Rozarija Mikić, Josip Pečarić, Mirna Rodić

**Affiliations:** Faculty of Textile Technology, University of Zagreb, Prilaz baruna Filipovića 28a, Zagreb, 10000 Croatia

**Keywords:** 26D15, 26A51, 26A24, Jensen’s inequality, converse Jensen’s inequality, Hermite-Hadamard’s inequality, Giaccardi’s inequality, Levinson’s inequality, Green function, mean-value theorems

## Abstract

We derive the Levinson type generalization of the Jensen and the converse Jensen inequality for real Stieltjes measure, not necessarily positive. As a consequence, also the Levinson type generalization of the Hermite-Hadamard inequality is obtained. Similarly, we derive the Levinson type generalization of Giaccardi’s inequality. The obtained results are then applied for establishing new mean-value theorems. The results from this paper represent a generalization of several recent results.

## Introduction and preliminary results

The well-known Jensen inequality asserts that for a convex function $\varphi \colon I\subseteq \mathbb{R}\to \mathbb{R}$ we have

1.1 $$ \varphi \Biggl(\frac{1}{P_{n}}\sum_{i=1}^{n} p_{i} x_{i} \Biggr)\leq \frac{1}{P _{n}}\sum _{i=1}^{n} p_{i} \varphi (x_{i}), $$

where $x_{i}\in I$ for $i=1,\ldots,n$, and $p_{i}$ are nonnegative real numbers such that $P_{n}=\sum_{i=1}^{n} p_{i}>0$.

Steffensen [1] showed that inequality (1.1) also holds in the case when $(x_{1},\ldots,x_{n})$ is a monotonic *n*-tuple of numbers from the interval *I* and $(p_{1},\ldots,p_{n})$ is an arbitrary real *n*-tuple such that $0\le P_{k}\le P_{n}$ ($k=1,\ldots,n$), $P_{n}>0$, where $P_{k}=\sum_{i=1}^{k}p_{i}$. His result is called the Jensen-Steffensen inequality.

Boas [2] gave the integral analog of the Jensen-Steffensen inequality.

### Theorem 1.1

[2]

*Let* $\varphi \colon I \to \mathbb{R}$ *be a continuous convex function*, *where* *I* *is the range of the continuous monotonic function* (*either increasing or decreasing*) $f\colon [a,b]\to \mathbb{R}$, *and let* $\lambda \colon [a,b]\to \mathbb{R}$ *be either continuous or of bounded variation satisfying*

$$\begin{aligned} \lambda (a)\le \lambda (x) \le \lambda (b) \quad \textit{for all } x\in [a,b], \lambda (a)< \lambda (b). \end{aligned}$$

*Then*

1.2 $$\begin{aligned} \varphi \biggl(\frac{\int_{a}^{b} f(x)\,d\lambda (x)}{ \int_{a}^{b}d\lambda (x)} \biggr)\le \frac{\int_{a}^{b} \varphi (f(x))\,d\lambda (x)}{ \int_{a}^{b}d\lambda (x)}. \end{aligned}$$

The generalization of this result is also given by Boas in [2]. It is the so-called Jensen-Boas inequality (see also [3]).

### Theorem 1.2

[2]

*If* $\lambda \colon [a,b]\to \mathbb{R}$ *is either continuous or of bounded variation satisfying*

$$\begin{aligned} \lambda (a)\le \lambda (x_{1})\le \lambda (y_{1})\le \lambda (x_{2}) \le \cdots \le \lambda (y_{n-1})\le \lambda (x_{n})\le \lambda (b) \end{aligned}$$

*for all* $x_{k}\in \langle y_{k-1},y_{k}\rangle $, $y_{0}=a$, $y_{n}=b$, *and* $\lambda (a)<\lambda (b)$, *and if* *f* *is continuous and monotonic* (*either increasing or decreasing*) *in each of the* *n* *intervals* $\langle y_{k-1},y_{k}\rangle $, *then inequality *(1.2) *holds*.

The following theorem states the well-known Levinson inequality.

### Theorem 1.3

[4]

*Let* $f\colon \langle 0,2c \rangle \to \mathbb{R}$ *satisfy* $f'''\ge 0$ *and let* $p_{i}, x_{i}, y_{i}$, $i=1,\ldots,n$ *be such that* $p_{i}>0$, $\sum_{i=1}^{n}p_{i}=1$, $0\le x_{i} \le c$ *and*

1.3 $$\begin{aligned} x_{1}+y_{1}=x_{2}+y_{2}= \cdots=x_{n}+y_{n}. \end{aligned}$$

*Then the following inequality is valid*:

1.4 $$\begin{aligned} \sum_{i=1}^{n}p_{i}f(x_{i})-f( \bar{x})\le \sum_{i=1}^{n}p_{i}f(y_{i})-f( \bar{y}), \end{aligned}$$

*where* $\bar{x}=\sum_{i=1}^{n}p_{i}x_{i}$ *and* $\bar{y}=\sum_{i=1}^{n}p _{i}y_{i}$ *denote the weighted arithmetic means*.

Numerous papers have been devoted to extensions and generalizations of this result, as well as to weakening the assumptions under which inequality (1.4) is valid (see for instance [5–8], and [9]).

A function $f\colon I\to \mathbb{R}$ is called *k*-convex if $[x_{0},\ldots,x_{k}]f\ge 0$ for all choices of $k+1$ distinct points $x_{0},x_{1},\ldots,x_{k}\in I$. If the *k*th derivative of a convex function exists, then $f^{(k)}\ge 0$, but $f^{(k)}$ may not exist (for properties of divided differences and *k*-convex functions see [3]).

### Remark 1.4

(i) Bullen [6] rescaled Levinson’s inequality to a general interval $[a,b]$ and showed that if function f is 3-convex and $p_{i}, x_{i}, y_{i}$, $i=1,\ldots,n$, are such that $p_{i}>0$, $\sum_{i=1} ^{n}p_{i}=1$, $a\le x_{i}$, $y_{i}\le b$, (1.3) holds for some $c\in \langle a,b\rangle $ and
  1.5 $$ \max \{x_{1},\ldots,x_{n}\}\le \max \{y_{1},\ldots,y_{n}\}, $$
  then (1.4) holds.
(ii) Pečarić [8] proved that inequality (1.4) is valid when one weakens the previous assumption (1.5) to
  $$ x_{i}+x_{n-i+1}\le 2c\quad\mbox{and}\quad\dfrac{p_{i}x_{i}+p_{n-i+1}x_{n-i+1}}{p_{i}+p_{n-i+1}}\le c, \quad \mbox{for } i=1,2,\ldots,n. $$
(iii) Mercer [7] made a significant improvement by replacing condition (1.3) with a weaker one, *i.e.* he proved that inequality (1.4) holds under the following conditions:
  1.6 $$\begin{aligned}& f'''\ge 0, \qquad p_{i}>0, \qquad \sum_{i=1}^{n}p_{i}=1, \qquad a\le x_{i},y_{i} \le b, \\& \max \{x_{1},\ldots,x_{n}\}\le \max \{y_{1}, \ldots,y_{n}\}, \\& \sum_{i=1}^{n}p_{i}(x_{i}- \bar{x})^{2}=\sum_{i=1}^{n}p_{i}(y_{i}- \bar{y})^{2}. \end{aligned}$$
(iv) Witkowski [9] showed that it is enough to assume that *f* is 3-convex in Mercer’s assumptions. Furthermore, Witkowski weakened the assumption (1.6) and showed that equality can be replaced by inequality in a certain direction.

Furthermore, Baloch, Pečarić, and Praljak in their paper [5] introduced a new class of functions $\mathcal{K}_{1} ^{c}(a,b)$ that extends 3-convex functions and can be interpreted as functions that are ‘3-convex at point $c\in \langle a,b\rangle $’. They showed that $\mathcal{K}_{1}^{c}(a,b)$ is the largest class of functions for which Levinson’s inequality (1.4) holds under Mercer’s assumptions, *i.e.* that $f\in \mathcal{K}_{1}^{c}(a,b)$ if and only if inequality (1.4) holds for arbitrary weights $p_{i}>0$, $\sum_{i=1}^{n}p_{i}=1$ and sequences $x_{i}$ and $y_{i}$ that satisfy $x_{i}\le c\le y_{i}$ for $i=1,2,\ldots,n$.

We give the definition of the class $\mathcal{K}_{1}^{c}(a,b)$ extended to an arbitrary interval *I*.

### Definition 1.5

*Let* $f\colon I \to \mathbb{R}$ *and* $c\in I^{\circ }$, *where* $I^{\circ }$ *is the interior of* *I*. *We say that* $f\in \mathcal{K} _{1}^{c}(I)$ ($f\in \mathcal{K}_{2}^{c}(I)$) *if there exists a constant* *D* *such that the function* $F(x)=f(x)-\frac{D}{2}x^{2}$ *is concave* (*convex*) *on* $\langle -\infty ,c]\cap I$ *and convex* (*concave*) *on* $[c,+\infty \rangle \cap I$.

### Remark 1.6

For the class $\mathcal{K}_{1}^{c}(a,b)$ the following useful results hold (see [5]):

(1): If $f \in \mathcal{K}_{i}^{c}(a,b)$, $i=1,2$, and $f''(c)$ exists, then $f''(c)=D$.
(2): The function $f:(a,b)\to \mathbb{R}$ is 3-convex (3-concave), if and only if $f \in \mathcal{K}_{1}^{c}(a,b)$ ($f \in \mathcal{K}_{2}^{c}(a,b)$) for every $c\in (a,b)$.

Jakšetić, Pečarić, and Praljak in [10] gave the following Levinson type generalization of the Jensen-Boas inequality.

### Theorem 1.7

[10]

*Let* $c\in I^{\circ }$ *and let* $f\colon [a_{1},b_{1}]\to \mathbb{R}$ *and* $g\colon [a_{2},b_{2}] \to \mathbb{R}$ *be continuous monotonic functions* (*either increasing or decreasing*) *with ranges* $I\cap \langle -\infty ,c]$ *and* $I\cap [c,+ \infty \rangle $, *respectively*. *Let* $\lambda \colon [a_{1},b_{1}] \to \mathbb{R}$ *and* $\mu \colon [a_{2},b_{2}]\to \mathbb{R}$ *be continuous or of bounded variation satisfying*

$$\begin{aligned} \lambda (a_{1})\le \lambda (x_{1})\le \lambda (y_{1})\le \lambda (x _{2})\le \cdots \le \lambda (y_{n-1})\le \lambda (x_{n})\le \lambda (b _{1}) \end{aligned}$$

*for all* $x_{k}\in \langle y_{k-1},y_{k}\rangle $, $y_{0}=a_{1}$, $y_{n}=b_{1}$, *and* $\lambda (a_{1})<\lambda (b_{1})$, *and*

$$\begin{aligned} \mu (a_{2})\le \mu (u_{1})\le \mu (v_{1})\le \lambda (u_{2})\le \cdots \le \mu (v_{n-1})\le \mu (u_{n})\le \mu (b_{2}) \end{aligned}$$

*for all* $u_{k}\in \langle v_{k-1},v_{k}\rangle $, $v_{0}=a_{2}$, $v_{n}=b_{2}$, *and* $\mu (a_{2})<\mu (b_{2})$. *If* $\varphi \in \mathcal{K}_{1}^{c}(I)$ *is continuous and if*

$$\begin{aligned}& \frac{\int_{a_{1}}^{b_{1}}f^{2}(x)\,d\lambda (x)}{\int_{a_{1}}^{b_{1}}d\lambda (x)}- \biggl( \frac{\int_{a_{1}}^{b_{1}}f(x)\,d\lambda (x)}{ \int_{a_{1}}^{b_{1}}d\lambda (x)} \biggr)^{2} \\& \quad = \frac{\int_{a_{2}}^{b _{2}}g^{2}(x)\,d\mu (x)}{\int_{a_{2}}^{b_{2}}d\mu (x)}- \biggl( \frac{ \int_{a_{2}}^{b_{2}}g(x)\,d\mu (x)}{\int_{a_{2}}^{b_{2}}d\mu (x)} \biggr)^{2} \end{aligned}$$

*holds*, *then*

1.7 $$\begin{aligned}& \frac{\int_{a_{1}}^{b_{1}}\varphi (f(x))\,d\lambda (x)}{\int_{a_{1}} ^{b_{1}}d\lambda (x)}-\varphi \biggl( \frac{\int_{a_{1}}^{b_{1}}f(x)\,d\lambda (x)}{\int_{a_{1}}^{b_{1}}d\lambda (x)} \biggr) \\& \quad \le \frac{ \int_{a_{2}}^{b_{2}}\varphi (g(x))\,d\mu (x)}{\int_{a_{2}}^{b_{2}}d\mu (x)}-\varphi \biggl( \frac{\int_{a_{2}}^{b_{2}}g(x)\,d\mu (x)}{ \int_{a_{2}}^{b_{2}}d\mu (x)} \biggr). \end{aligned}$$

On the other hand, in [11] Pečarić, Perić, and Rodić Lipanović generalized the Jensen inequality (1.2) for a real Stieltjes measure. They considered the Green function *G* defined on $[\alpha , \beta ]\times [\alpha , \beta ]$ by

1.8 $$ G(t,s)= \textstyle\begin{cases} \frac{(t-\beta )(s-\alpha )}{\beta -\alpha } &\text{for } \alpha \leq s\leq t, \\ \frac{(s-\beta )(t-\alpha )}{\beta -\alpha } &\text{for } t\leq s \leq \beta , \end{cases} $$

which is convex and continuous with respect to both *s* and *t*. The function *G* is continuous under *s* and continuous under *t*, and it can easily be shown by integrating by parts that any function $\varphi :[\alpha , \beta ]\rightarrow \mathbb{R}$, $\varphi \in C ^{2}([\alpha , \beta ])$, can be represented by

1.9 $$ \varphi (x)=\frac{\beta -x}{\beta -\alpha }\varphi (\alpha )+ \frac{x- \alpha }{\beta -\alpha }\varphi (\beta )+ \int_{\alpha }^{\beta} G(x,s) \varphi ''(s)\,ds. $$

Using that fact, the authors in [11] gave the conditions under which inequality (1.2) holds for a real Stieltjes measure, which is not necessarily positive nor increasing. This result is stated in the following theorem.

### Theorem 1.8

[11]

*Let* $g:[a,b]\rightarrow \mathbb{R}$ *be continuous function and* $[\alpha , \beta ]$ *interval such that the image of* *g* *is a subset of* $[\alpha , \beta ]$. *Let* $\lambda :[a,b]\rightarrow \mathbb{R}$ *be continuous function or the function of bounded variation*, *such that* $\lambda (a)\neq \lambda (b)$ *and*

$$\begin{aligned} \frac{\int_{a}^{b} g(x)\,d\lambda (x)}{\int_{a}^{b}d\lambda (x)}\in [ \alpha , \beta ]. \end{aligned}$$

*Then the following two statements are equivalent*:

(1): *For every continuous convex function* $\varphi : [\alpha, \beta ]\rightarrow \mathbb{R}$
  1.10 $$ \varphi \biggl( \frac{\int_{a}^{b} g(x)\,d\lambda (x)}{\int_{a}^{b}d\lambda (x)} \biggr) \leq \frac{\int_{a}^{b} \varphi ( g(x) )\,d\lambda (x)}{\int_{a}^{b}d\lambda (x)} $$
  *holds*.
(2): *For all* $s\in [\alpha , \beta ]$
  1.11 $$ G \biggl( \frac{\int_{a}^{b} g(x)\,d\lambda (x)}{\int_{a}^{b}d\lambda (x)},s \biggr) \leq \frac{\int_{a}^{b} G ( g(x),s )\,d\lambda (x)}{\int_{a}^{b}d\lambda (x)} $$
  *holds*, *where the function* $G: [\alpha , \beta ]\times [\alpha , \beta ]\rightarrow \mathbb{R}$ *is defined in* (1.8).

*Furthermore*, *the statements* (1) *and* (2) *are also equivalent if we change the sign of inequality in both* (1.10) *and* (1.11).

Note that for every continuous concave function $\varphi : [\alpha , \beta ]\rightarrow \mathbb{R}$ inequality (1.10) is reversed, *i.e.* the following corollary holds.

### Corollary 1.9

[11]

*Let the conditions from the previous theorem hold*. *Then the following two statements are equivalent*:

(1′): *For every continuous concave function* $\varphi : [ \alpha , \beta ]\rightarrow \mathbb{R}$ *the reverse inequality in *(1.10) *holds*.
(2′): *For all* $s\in [\alpha , \beta ]$ *inequality* (1.11) *holds*, *where the function* *G* *is defined in* (1.8).

*Moreover*, *the statements* (1′) *and* (2′) *are also equivalent if we change the sign of inequality in both statements* (1′) *and* (2′).

The main aim of our paper is to give a Levinson type generalization of the result from Theorem 1.8. In that way, a generalization of Theorem 1.7 for real Stieltjes measure, not necessarily positive nor increasing, will also be obtained.

## Main results

In order to simplify the notation, throughout this paper we use the following notation:

$$\begin{aligned} \bar{f}=\frac{\int_{a_{1}}^{b_{1}} f(x)\,d\lambda (x)}{\int_{a_{1}}^{b_{1}}d\lambda (x)}\quad \mbox{and}\quad \bar{g}=\frac{\int_{a_{2}}^{b_{2}} g(x)\,d\mu (x)}{\int_{a_{2}}^{b_{2}}d\mu (x)}. \end{aligned}$$

The following theorem states our main result.

### Theorem 2.1

*Let* $f\colon [a_{1},b_{1}]\to \mathbb{R}$ *and* $g\colon [a_{2},b_{2}] \to \mathbb{R}$ *be continuous functions*, $[\alpha , \beta ]\subseteq \mathbb{R}$ *an interval and* $c\in \langle \alpha ,\beta \rangle $ *such that* $f([a_{1},b_{1}])\subseteq [\alpha , c]$ *and* $g([a_{2},b_{2}]) \subseteq [c,\beta ]$. *Let* $\lambda \colon [a_{1},b_{1}]\to \mathbb{R}$ *and* $\mu \colon [a_{2},b_{2}]\to \mathbb{R}$ *be continuous functions or functions of bounded variation such that* $\lambda (a_{1}) \neq \lambda (b_{1})$ *and* $\mu (a_{2})\neq \mu (b_{2})$ *and such that*

$$\begin{aligned} \bar{f}\in [\alpha ,c]\quad \textit{and}\quad \bar{g}\in [c,\beta ] \end{aligned}$$

*and*

2.1 $$\begin{aligned} C:=\frac{\int_{a_{1}}^{b_{1}} f^{2}(x)\,d\lambda (x)}{\int_{a_{1}}^{b_{1}}d\lambda (x)}-\bar{f}^{2}=\frac{\int_{a_{2}}^{b_{2}} g^{2}(x)\,d\mu (x)}{\int_{a_{2}}^{b_{2}}d\mu (x)}-\bar{g}^{2} \end{aligned}$$

*holds*.

*If for all* $s_{1}\in [\alpha ,c]$ *and for all* $s_{2}\in [c,\beta ]$ *we have*

2.2 $$\begin{aligned} G(\bar{f},s_{1})\le \frac{\int_{a_{1}}^{b_{1}} G(f(x),s_{1})\,d\lambda (x)}{ \int_{a_{1}}^{b_{1}}d\lambda (x)}\quad \textit{and}\quad G( \bar{g},s_{2})\le \frac{\int_{a_{2}}^{b_{2}} G(g(x),s_{2})\,d\mu (x)}{ \int_{a_{2}}^{b_{2}}d\mu (x)}, \end{aligned}$$

*where the function* *G* *is defined in* (1.8), *then for every continuous function* $\varphi \in \mathcal{K}_{1}^{c}([\alpha ,\beta ])$ *we have*

2.3 $$\begin{aligned} \frac{\int_{a_{1}}^{b_{1}} \varphi (f(x))\,d\lambda (x)}{\int_{a_{1}} ^{b_{1}}d\lambda (x)}-\varphi (\bar{f})\le \frac{D}{2}C \le \frac{ \int_{a_{2}}^{b_{2}} \varphi (g(x))\,d\mu (x)}{\int_{a_{2}}^{b_{2}}d\mu (x)}- \varphi (\bar{g}). \end{aligned}$$

*The statement also holds if we reverse all signs of inequalities in *(2.2) *and *(2.3).

### Proof

Let $\varphi \in \mathcal{K}_{1}^{c}([\alpha ,\beta ])$ be continuous function on $[\alpha ,\beta ]$ and let $\phi (x)=\varphi (x)-\frac{D}{2}x^{2}$, where *D* is the constant from Definition 1.5.

Since the function *ϕ* is continuous and concave on $[\alpha ,c]$ and for all $s_{1}\in [\alpha ,c]$ (2.2) holds, from Corollary 1.9 it follows that

$$\begin{aligned} \phi (\bar{f})\ge \frac{\int_{a_{1}}^{b_{1}} \phi (f(x))\,d\lambda (x)}{ \int_{a_{1}}^{b_{1}}d\lambda (x)}. \end{aligned}$$

When we rearrange the previous inequality, we get

2.4 $$\begin{aligned} \frac{\int_{a_{1}}^{b_{1}} \varphi (f(x))\,d\lambda (x)}{\int_{a_{1}} ^{b_{1}}d\lambda (x)}-\varphi (\bar{f}) \le \frac{D}{2} \biggl[ \frac{ \int_{a_{1}}^{b_{1}} f^{2}(x)\,d\lambda (x)}{\int_{a_{1}}^{b_{1}}d\lambda (x)}-\bar{f}^{2} \biggr]. \end{aligned}$$

Since the function *ϕ* is continuous and convex on $[c,\beta ]$ and for all $s_{2}\in [c,\beta ]$ (2.2) holds, from Theorem 1.8 it follows that

$$\begin{aligned} \phi ( \bar{g} ) \le \frac{\int_{a_{2}}^{b_{2}} \phi ( g(x) )\,d\mu (x)}{\int_{a_{2}}^{b_{2}}d\mu (x)}, \end{aligned}$$

and after rearranging we get

2.5 $$\begin{aligned} \frac{D}{2} \biggl[\frac{\int_{a_{2}}^{b_{2}} g^{2}(x)\,d\mu (x)}{\int _{a_{2}}^{b_{2}}d\mu (x)}-\bar{g}^{2} \biggr] \le \frac{\int_{a_{2}} ^{b_{2}} \varphi ( g(x) )\,d\mu (x)}{\int_{a_{2}}^{b_{2}}d\mu (x)}-\varphi ( \bar{g} ) . \end{aligned}$$

Inequality (2.3) follows directly by combining inequalities (2.4) and (2.5), and taking into account the condition (2.1). □

### Corollary 2.2

*Let the conditions from the previous theorem hold*.

(i) *If for all* $s_{1}\in [\alpha , c]$ *and* $s_{2}\in [c,\beta ]$ *inequalities* (2.2) *hold*, *where the function* *G* *is defined in* (1.8), *then for every continuous function* $\varphi \in \mathcal{K}_{2}^{c}([\alpha ,\beta ])$ *the reverse inequalities in* (2.3) *hold*.
(ii) *If for all* $s_{1}\in [\alpha , c]$ *and* $s_{2}\in [c,\beta ]$ *the reverse inequalities in* (2.2) *hold*, *then for every continuous function* $\varphi \in \mathcal{K}_{2}^{c}([\alpha ,\beta ])$ (2.3) *holds*.

### Remark 2.3

It is obvious from the proof of the previous theorem that if we replace the equality (2.1) by a weaker condition

2.6 $$\begin{aligned} C_{1}:= \frac{D}{2} \biggl[\frac{\int_{a_{1}}^{b_{1}} f^{2}(x)\,d\lambda (x)}{ \int_{a_{1}}^{b_{1}}d\lambda (x)}- \bar{f}^{2} \biggr] \le C_{2}:= \frac{D}{2} \biggl[ \frac{\int_{a_{2}}^{b_{2}} g^{2}(x)\,d\mu (x)}{\int _{a_{2}}^{b_{2}}d\mu (x)}-\bar{g}^{2} \biggr], \end{aligned}$$

then (2.3) becomes

$$\begin{aligned} \frac{\int_{a_{1}}^{b_{1}} \varphi (f(x))\,d\lambda (x)}{\int_{a_{1}} ^{b_{1}}d\lambda (x)}-\varphi (\bar{f})\le C_{1}\le C_{2} \le \frac{ \int_{a_{2}}^{b_{2}} \varphi (g(x))\,d\mu (x)}{\int_{a_{2}}^{b_{2}}d\mu (x)}- \varphi (\bar{g}). \end{aligned}$$

Since the function *φ* belongs to class $\mathcal{K}_{1}^{c}([ \alpha ,\beta ])$, we have $\varphi_{-}''(c)\le D\le \varphi_{+}''(c)$ (see [5]), so if additionally *φ* is convex (resp. concave), the condition (2.6) can be further weakened to

$$\begin{aligned} \frac{\int_{a_{1}}^{b_{1}} f^{2}(x)\,d\lambda (x)}{\int_{a_{1}}^{b_{1}}d\lambda (x)}-\bar{f}^{2} \le \frac{\int_{a_{2}}^{b_{2}} g^{2}(x)\,d\mu (x)}{\int_{a_{2}}^{b_{2}}d\mu (x)}- \bar{g}^{2}. \end{aligned}$$

### Remark 2.4

It is easy to see that Theorem 2.1 further generalizes the Levinson type generalization of the Jensen-Boas inequality given in Theorem 1.7. Namely, if in Theorem 2.1 we set the functions *f* and *g* to be monotonic, and the functions *λ* and *μ* to satisfy

$$\begin{aligned} \lambda (a_{1})\le \lambda (x_{1})\le \lambda (y_{1})\le \lambda (x _{2})\le \cdots \le \lambda (y_{n-1})\le \lambda (x_{n})\le \lambda (b _{1}) \end{aligned}$$

for all $x_{k}\in \langle y_{k-1},y_{k}\rangle $, $y_{0}=a_{1}$, $y_{n}=b_{1}$, and $\lambda (a_{1})<\lambda (b_{1})$, and

$$\begin{aligned} \mu (a_{2})\le \mu (u_{1})\le \mu (v_{1})\le \lambda (u_{2})\le \cdots \le \mu (v_{n-1})\le \mu (u_{n})\le \mu (b_{2}) \end{aligned}$$

for all $u_{k}\in \langle v_{k-1},v_{k}\rangle $, $v_{0}=a_{2}$, $v_{n}=b_{2}$, and $\mu (a_{2})<\mu (b_{2})$, then since the function *G* is continuous and convex in both variables, we can apply the Jensen inequality and see that for all $s_{1}\in [\alpha , c]$ and $s_{2}\in [c,\beta ]$ inequalities (2.2) hold, so we get exactly Theorem 1.7.

## Discrete case

In this section we give the results for the discrete case. The proofs are similar to those in the integral case given in the previous section, so we will state these results without the proofs.

In Levinson’s inequality (1.3) and its generalizations (see [5]) we see that $p_{i}$ ($i=1,\ldots,n$) are positive real numbers. Here, we will give a generalization of that result, allowing $p_{i}$ to also be negative, with the sum not equal to zero, but with a supplementary demand on $p_{i}$ and $x_{i}$ given by using the Green function *G* defined in (1.8).

Here we use the common notation: for real *n*-tuples $(x_{1},\ldots,x_{n})$ and $(p_{1},\ldots,p_{n})$ we set $P_{k}=\sum_{i=1}^{k}p_{i}$, $\bar{P_{k}}=P_{n}-P_{k-1}$ ($k=1,\ldots,n$) and $\bar{x}= \frac{1}{P_{n}}\sum_{i=1}^{n}p_{i}x_{i}$. Analogously, for real *m*-tuples $(y_{1},\ldots,y_{m})$ and $(q_{1},\ldots,q_{m})$ we define $Q_{k}$, $\bar{Q_{k}}$ ($k=1,\ldots,m$) and *ȳ*.

We already know from the first section that we can represent any function $\varphi :[\alpha , \beta ]\rightarrow \mathbb{R}$, $\varphi \in C^{2} ( [\alpha , \beta ] ) $, in the form (1.9), where the function *G* is defined in (1.8), and by some calculation it is easy to show that the following holds:

$$ \varphi (\bar{x})-\frac{1}{P_{n}} \sum _{i=1}^{n} p_{i} \varphi (x_{i}) = \int_{\alpha }^{\beta} \Biggl( G(\bar{x},s)- \frac{1}{P_{n}} \sum_{i=1} ^{n} p_{i} G(x_{i},s) \Biggr) \varphi ''(s) \,ds. $$

Using that fact the authors in [11] derived the analogous results of Theorem 1.8 and Corollary 1.9 for discrete case, and here, similarly as in the previous section, we get the following results.

### Theorem 3.1

*Let* $[\alpha , \beta ]\subseteq \mathbb{R}$ *be an interval and* $c\in \langle \alpha ,\beta \rangle $. *Let* $x_{i}\in [a_{1},b_{1}] \subseteq [\alpha ,c]$, $p_{i}\in \mathbb{R}$ ($i=1,\ldots,n$) *be such that* $P_{n}\neq 0$ *and* $\bar{x}\in [\alpha ,c]$, *and let* $y_{j}\in [a_{2},b _{2}]\subseteq [c, \beta ]$, $q_{j}\in \mathbb{R}$ ($j=1,\ldots,m$) *be such that* $Q_{m}\neq 0$ *and* $\bar{y}\in [c,\beta ]$ *and let*

3.1 $$\begin{aligned} C:=\frac{1}{P_{n}}\sum_{i=1}^{n}p_{i}x_{i}^{2}-\bar{x}^{2} =\frac{1}{Q_{m}}\sum_{j=1}^{m}q_{j}y_{j}^{2}- \bar{y}^{2}. \end{aligned}$$

*If for all* $s_{1}\in [\alpha ,c]$ *and for all* $s_{2}\in [c,\beta ]$ *we have*

3.2 $$\begin{aligned} G(\bar{x},s_{1})\le \frac{1}{P_{n}}\sum _{i=1}^{n}p_{i}G(x_{i},s_{1}) \quad \textit{and}\quad G(\bar{y},s_{2})\le \frac{1}{Q_{m}}\sum _{j=1}^{m}q_{j}G(y_{j},s_{2}), \end{aligned}$$

*where the function* *G* *is defined in* (1.8), *then for every continuous function* $\varphi \in \mathcal{K}_{1}^{c}([\alpha ,\beta ])$ *we have*

3.3 $$\begin{aligned} \frac{1}{P_{n}}\sum_{i=1}^{n}p_{i} \varphi (x_{i})-\varphi (\bar{x}) \le \frac{D}{2}C \le \frac{1}{Q_{m}}\sum_{j=1}^{m}q_{j} \varphi (y_{j})- \varphi (\bar{y}), \end{aligned}$$

*where* *D* *is the constant from Definition* 1.5.

*Inequality* (3.3) *is reversed if we change the signs of inequalities in* (3.2).

### Corollary 3.2

*Let the conditions from the previous theorem hold*.

(i) *If for all* $s_{1}\in [\alpha , c]$ *and* $s_{2}\in [c,\beta ]$ *the inequalities in* (3.2) *hold*, *where the function* *G* *is defined in* (1.8), *then for every continuous function* $\varphi \in \mathcal{K}_{2}^{c}([\alpha ,\beta ])$ *the reverse inequalities in* (3.3) *hold*.
(ii) *If for all* $s_{1}\in [\alpha , c]$ *and* $s_{2}\in [c, \beta ]$ *the reversed inequalities in* (3.2) *hold*, *then for every continuous function* $\varphi \in \mathcal{K}_{2}^{c}([ \alpha ,\beta ])$ (3.3) *holds*.

### Remark 3.3

Theorem 3.1 is the generalization of Levinson’s type inequality given in [5]. Namely, since the function *G* is convex in both variables, in the case when all $p_{i}>0$ and $q_{j}>0$ we can apply the Jensen inequality and we see that for all $s_{1}\in [\alpha , c]$ and $s_{2}\in [c,\beta ]$ inequalities (3.2) hold. Now from Theorem 3.1 and Corollary 3.2 we get the result from [5].

### Remark 3.4

We can replace the equality from the condition (3.1) by a weaker condition in the analogous way as in Remark 2.3 from the previous section.

## Converses of the Jensen inequality

The Jensen inequality for convex functions implies a whole series of other classical inequalities. One of the most famous ones amongst them is the so-called Edmundson-Lah-Ribarič inequality which states that, for a positive measure *μ* on $[0,1]$ and a convex function *ϕ* on $[m,M]$ ($-\infty < m< M<+\infty $), if *f* is any *μ*-measurable function on $[0,1]$ such that $m\le f(x)\le M$ for $x\in [0,1]$, one has

4.1 $$\begin{aligned} \dfrac{\int_{0}^{1}\phi (f)\,d\mu }{\int_{0}^{1}d\mu }\le \dfrac{M- \bar{f}}{M-m}\phi (m)+\dfrac{\bar{f}-m}{M-m}\phi (M), \end{aligned}$$

where $\bar{f}=\int_{0}^{1}f\,d\mu / \int_{0}^{1}d\mu $.

It was obtained in 1973. by Lah and Ribarič in their paper [12]. Since then, there have been many papers written on the subject of its generalizations and converses (for instance, see [13] and [3]).

In [14] the authors gave a Levinson type generalization of inequality (4.1) for positive measures. In this section we will obtain a similar result involving signed measures, with a supplementary demand by using the Green function *G* defined in (1.8). In order to do so, we first need to state a result from [11], which gives us a version of the Edmundson-Lah-Ribarič inequality for signed measures.

### Theorem 4.1

[11]

*Let* $g:[a,b]\rightarrow \mathbb{R}$ *be continuous function and* $[\alpha , \beta ]$ *be an interval such that the image of* *g* *is a subset of* $[\alpha , \beta ]$. *Let* $m, M \in [\alpha , \beta ]$ $(m\neq M)$ *be such that* $m\leq g(t) \leq M$ *for all* $t\in [a,b]$. *Let* $\lambda :[a,b]\rightarrow \mathbb{R}$ *be continuous function or the function of bounded variation*, *and* $\lambda (a)\neq \lambda (b)$. *Then the following two statements are equivalent*:

(1): *For every continuous convex function* $\varphi : [\alpha, \beta ]\rightarrow \mathbb{R}$
  4.2 $$ \frac{\int_{a}^{b} \varphi ( g(x) )\,d\lambda (x)}{\int_{a} ^{b}d\lambda (x)}\leq \frac{M-\bar{g}}{M-m}\varphi (m)+ \frac{\bar{g}-m}{M-m}\varphi (M) $$
  *holds*, *where* $\bar{g}=\frac{\int_{a}^{b} g(x)\,d\lambda (x)}{\int_{a} ^{b}d\lambda (x)}$.
(2): *For all* $s\in [\alpha , \beta ]$
  4.3 $$ \frac{\int_{a}^{b} G ( g(x),s )\,d\lambda (x)}{\int_{a}^{b}d\lambda (x)}\leq \frac{M-\bar{g}}{M-m}G(m,s)+ \frac{\bar{g}-m}{M-m}G(M,s) $$
  *holds*, *where the function* $G: [\alpha , \beta ]\times [\alpha , \beta ]\rightarrow \mathbb{R}$ *is defined in* (1.8).

*Furthermore*, *the statements* $(1)$ *and* $(2)$ *are also equivalent if we change the sign of inequality in both* (4.2) *and* (4.3).

Note that for every continuous concave function $\varphi : [\alpha , \beta ]\rightarrow \mathbb{R}$ inequality (4.2) is reversed, *i.e.* the following corollary holds.

### Corollary 4.2

[11]

*Let the conditions from the previous theorem hold*. *Then the following two statements are equivalent*:

(1′): *For every continuous concave function* $\varphi : [\alpha , \beta ]\rightarrow \mathbb{R}$ *the reverse inequality in* (4.2) *holds*.
(2′): *For all* $s\in [\alpha , \beta ]$ *inequality* (4.3) *holds*, *where the function* *G* *is defined in *(1.8).

*Moreover*, *the statements* (1′) *and* (2′) *are also equivalent if we change the sign of inequality in both statements* (1′) *and* (2′).

In the following theorem we give the Levinson type generalization of the upper result, and we use a similar method to Section 2 of this paper.

### Theorem 4.3

*Let* $f\colon [a_{1},b_{1}]\to \mathbb{R}$ *and* $g\colon [a_{2},b_{2}] \to \mathbb{R}$ *be continuous functions*, $[\alpha , \beta ]\subseteq \mathbb{R}$ *an interval and* $c\in \langle \alpha ,\beta \rangle $ *such that* $f([a_{1},b_{1}])=[m_{1},M_{1}]\subseteq [\alpha , c]$ *and* $g([a_{2},b_{2}])=[m_{2},M_{2}]\subseteq [c,\beta ]$, *where* $m_{1}\neq M_{1}$ *and* $m_{2}\neq M_{2}$. *Let* $\lambda \colon [a_{1},b _{1}]\to \mathbb{R}$ *and* $\mu \colon [a_{2},b_{2}]\to \mathbb{R}$ *be continuous functions or functions of bounded variation such that* $\lambda (a_{1})\neq \lambda (b_{1})$ *and* $\mu (a_{2})\neq \mu (b_{2})$ *and*

4.4 $$\begin{aligned} C&:=\frac{M_{1}-\bar{f}}{M_{1}-m_{1}}m_{1}^{2}+\frac{\bar{f}-m_{1}}{M _{1}-m_{1}}M_{1}^{2}- \frac{\int_{a_{1}}^{b_{1}} f^{2}(x)\,d\lambda (x)}{ \int_{a_{1}}^{b_{1}}d\lambda (x)} \\ &=\frac{M_{2}-\bar{g}}{M_{2}-m_{2}}m_{2}^{2}+\frac{\overline{g}-m _{2}}{M_{2}-m_{2}}M_{2}^{2}- \frac{\int_{a_{2}}^{b_{2}} g^{2}(x)\,d\mu (x)}{\int_{a_{2}}^{b_{2}}d\mu (x)}. \end{aligned}$$

*If for all* $s\in [\alpha ,c]$ *we have*

4.5 $$ \frac{\int_{a_{1}}^{b_{1}}G ( f(x),s )\,d\lambda (x)}{\int_{a _{1}}^{b_{1}}d\lambda (x)}\le \frac{M_{1}-\bar{f}}{M_{1}-m_{1}}G(m _{1},s)+ \frac{\bar{f}-m_{1}}{M_{1}-m_{1}}G(M_{1},s) $$

*and for all* $s\in [c,\beta ]$ *we have*

4.6 $$ \frac{\int_{a_{2}}^{b_{2}}G ( g(x),s )\,d\mu (x)}{\int_{a_{2}} ^{b_{2}}d\mu (x)}\le \frac{M_{2}-\bar{g}}{M_{2}-m_{2}}G(m_{2},s)+ \frac{ \bar{g}-m_{2}}{M_{2}-m_{2}}G(M_{2},s), $$

*where the function* *G* *is defined in* (1.8), *then for every continuous function* $\varphi \in \mathcal{K}_{1}^{c}([\alpha ,\beta ])$ *we have*

4.7 $$\begin{aligned}& \frac{M_{1}-\bar{f}}{M_{1}-m_{1}} \varphi (m_{1})+\frac{\bar{f}-m_{1}}{M _{1}-m_{1}}\varphi (M_{1})-\frac{\int_{a_{1}}^{b_{1}} \varphi ( f(x) )\,d\lambda (x)}{\int_{a_{1}}^{b_{1}}d\lambda (x)} \\& \quad \le \frac{D}{2}C \le\frac{M_{2}-\bar{g}}{M_{2}-m_{2}}\varphi (m_{2})+\frac{\overline{g}-m _{2}}{M_{2}-m_{2}}\varphi (M_{2})-\frac{\int_{a_{2}}^{b_{2}}\varphi ( g(x) )\,d\mu (x)}{\int_{a_{2}}^{b_{2}}d\mu (x)}. \end{aligned}$$

*The statement also holds if we reverse all signs of inequalities in* (4.5), (4.6) *and* (4.7).

### Proof

Let $\varphi \in \mathcal{K}_{1}^{c}([\alpha ,\beta ])$ be continuous function on $[\alpha ,\beta ]$ and let $\phi (x)=\varphi (x)-\frac{D}{2}x^{2}$, where *D* is the constant from Definition 1.5.

Since the function *ϕ* is continuous and concave on $[\alpha ,c]$ and for all $s\in [\alpha ,c]$ (4.5) holds, from Corollary 4.2 it follows that

$$\begin{aligned} \frac{\int_{a_{1}}^{b_{1}} \phi ( f(x) )\,d\lambda (x)}{ \int_{a_{1}}^{b_{1}}d\lambda (x)}\ge \frac{M_{1}-\bar{f}}{M_{1}-m _{1}}\phi (m_{1})+ \frac{\bar{f}-m_{1}}{M_{1}-m_{1}}\phi (M_{1}). \end{aligned}$$

When we rearrange the previous inequality, we get

4.8 $$\begin{aligned}& \frac{M_{1}-\bar{f}}{M_{1}-m_{1}} \varphi (m_{1})+\frac{\bar{f}-m_{1}}{M _{1}-m_{1}}\varphi (M_{1})-\frac{\int_{a_{1}}^{b_{1}} \varphi ( f(x) )\,d\lambda (x)}{\int_{a_{1}}^{b_{1}}d\lambda (x)} \\& \quad \le \dfrac{D}{2} \biggl[ \frac{M_{1}-\bar{f}}{M_{1}-m_{1}}m_{1}^{2}+ \frac{ \bar{f}-m_{1}}{M_{1}-m_{1}}M_{1}^{2}-\frac{\int_{a_{1}}^{b_{1}} f^{2}(x)\,d\lambda (x)}{\int_{a_{1}}^{b_{1}}d\lambda (x)} \biggr] . \end{aligned}$$

Since the function *ϕ* is continuous and convex on $[c,\beta ]$ and for all $s\in [c,\beta ]$ (4.6) holds, from Theorem 4.1 it follows that

$$\begin{aligned} \frac{\int_{a_{2}}^{b_{2}} \phi ( g(x) )\,d\mu (x)}{\int_{a _{2}}^{b_{2}}d\mu (x)}\le \frac{M_{2}-\bar{g}}{M_{2}-m_{2}}\phi (m _{2})+ \frac{\bar{g}-m_{2}}{M_{2}-m_{2}}\phi (M_{2}), \end{aligned}$$

and after rearranging we get

4.9 $$\begin{aligned} \frac{D}{2} & \biggl[ \frac{M_{2}-\bar{g}}{M_{2}-m_{2}}m_{2}^{2}+ \frac{ \bar{g}-m_{2}}{M_{2}-m_{2}}M_{2}^{2}-\frac{\int_{a_{2}}^{b_{2}} g^{2}(x)\,d\mu (x)}{\int_{a_{2}}^{b_{2}}d\mu (x)} \biggr] \\ & \le \frac{M_{2}-\bar{g}}{M_{2}-m_{2}}\varphi (m_{2})+\frac{\bar{g}-m _{2}}{M_{2}-m_{2}}\varphi (M_{2})-\frac{\int_{a_{2}}^{b_{2}} \varphi ( g(x) )\,d\mu (x)}{\int_{a_{2}}^{b_{2}}d\mu (x)}. \end{aligned}$$

Inequality (4.7) follows directly by combining inequalities (4.8) and (4.9), and taking into account the condition (4.4). □

### Corollary 4.4

*Let the conditions from the previous theorem hold*.

(i) *If for all* $s\in [\alpha , c]$ *the inequality in* (4.5) *holds*, *and for all* $s\in [c, \beta ]$ *the inequality in* (4.6) *holds*, *then for every continuous function* $\varphi \in \mathcal{K}_{2}^{c}([\alpha ,\beta ])$ *the reverse inequalities in* (4.7) *hold*.
(ii) *If for all* $s\in [\alpha , c]$ *the reversed inequality in* (4.5) *holds*, *and for all* $s\in [c, \beta ]$ *the reversed inequality in* (4.6) *holds*, *then for every continuous function* $\varphi \in \mathcal{K}_{2}^{c}([\alpha ,\beta ])$ *the inequalities in* (4.7) *hold*.

### Remark 4.5

It is obvious from the proof of the previous theorem that if we replace the equality (4.4) by a weaker condition

4.10 $$\begin{aligned}& C_{1}: = \frac{D}{2} \biggl[ \frac{M_{1}-\bar{f}}{M_{1}-m_{1}}m_{1}^{2}+ \frac{ \bar{f}-m_{1}}{M_{1}-m_{1}}M_{1}^{2}-\frac{\int_{a_{1}}^{b_{1}} f^{2}(x)\,d\lambda (x)}{\int_{a_{1}}^{b_{1}}d\lambda (x)} \biggr] \\& \quad \le C_{2}:=\frac{D}{2} \biggl[\frac{M_{2}-\bar{g}}{M_{2}-m_{2}}m_{2} ^{2}+\frac{\overline{g}-m_{2}}{M_{2}-m_{2}}M_{2}^{2}-\frac{\int_{a _{2}}^{b_{2}} g^{2}(x)\,d\mu (x)}{\int_{a_{2}}^{b_{2}}d\mu (x)} \biggr], \end{aligned}$$

then (4.7) becomes

$$\begin{aligned}& \frac{M_{1}-\bar{f}}{M_{1}-m_{1}} \varphi (m_{1})+\frac{\bar{f}-m_{1}}{M_{1}-m_{1}}\varphi (M_{1})-\frac{\int_{a_{1}}^{b_{1}} \varphi ( f(x) )\,d\lambda (x)}{\int_{a_{1}}^{b_{1}}d\lambda (x)} \\& \quad \le C_{1} \le C_{2}\le \frac{M_{2}-\bar{g}}{M_{2}-m_{2}}\varphi (m_{2})+\frac{\overline{g}-m_{2}}{M_{2}-m_{2}}\varphi (M_{2})-\frac{\int_{a_{2}}^{b_{2}}\varphi ( g(x) )\,d\mu (x)}{\int_{a_{2}}^{b_{2}}d\mu (x)}. \end{aligned}$$

Since $\varphi_{-}''(c)\le D\le \varphi_{+}''(c)$ (see [5]), if additionally *φ* is convex (resp. concave), the condition (4.10) can be further weakened to

$$\begin{aligned}& \frac{M_{1}-\bar{f}}{M_{1}-m_{1}} m_{1}^{2}+\frac{\bar{f}-m_{1}}{M _{1}-m_{1}}M_{1}^{2}- \frac{\int_{a_{1}}^{b_{1}} f^{2}(x)\,d\lambda (x)}{ \int_{a_{1}}^{b_{1}}d\lambda (x)} \\& \quad \le \frac{M_{2}-\bar{g}}{M_{2}-m_{2}}m_{2}^{2}+\frac{\overline{g}-m_{2}}{M_{2}-m_{2}}M_{2}^{2}-\frac{\int_{a_{2}}^{b_{2}} g^{2}(x)\,d\mu (x)}{\int_{a_{2}}^{b_{2}}d\mu (x)} . \end{aligned}$$

## Discrete form of the converses of the Jensen inequality

In this section we give the Levinson type generalization for converses of Jensen’s inequality in discrete case. The proofs are similar to those in the integral case given in the previous section, so we give these results with the proofs omitted.

As we can represent any function $\varphi \colon [\alpha , \beta ] \to \mathbb{R}$, $\varphi \in C^{2} ( [\alpha , \beta ] ) $, in the form (1.9), where the function *G* is defined in (1.8), by some calculation it is easy to show that the following holds:

$$\begin{aligned}& \frac{1}{P_{n}}\sum_{i=1}^{n} p_{i} \varphi (x_{i})- \frac{b- \bar{x}}{b-a}\varphi (a)- \frac{\bar{x}-a}{b-a}\varphi (b) \\& \quad = \int_{\alpha }^{\beta} \Biggl( \frac{1}{P_{n}}\sum _{i=1}^{n} p_{i} G(x _{i},s)- \frac{b-\bar{x}}{b-a}G(a,s)-\frac{\bar{x}-a}{b-a}G(b,s) \Biggr) \varphi ''(s)\,ds. \end{aligned}$$

Using that fact the authors in [11] derived analogous results of Theorem 4.1 and Corollary 4.2 for discrete case.

In [14] the authors obtained the following Levinson type generalization of the discrete Edmundson-Lah-Ribarič inequality.

### Theorem 5.1

[14]

*Let* $-\infty < a\le A\le c \le b \le B <+\infty $. *If* $x_{i}\in [a,A]$, $y_{j}\in [b,B]$, $p_{i}>0$, $q_{j}>0$ *for* $i=1,\ldots,n$ *and* $j=1,\ldots,m$ *are such that* $\sum_{i=1}^{n}p_{i}=\sum_{j=1}^{m}q_{j}=1$ *and*

$$\begin{aligned} \dfrac{A-\bar{x}}{A-a}a^{2}+\dfrac{\bar{x}-a}{A-a}A^{2}-\sum _{i=1} ^{n}p_{i}x_{i}^{2} &=\dfrac{B-\bar{y}}{B-b}b^{2}+ \dfrac{\bar{y}-b}{B-b}B^{2}-\sum _{j=1}^{m}q_{j}y_{j}^{2}, \end{aligned}$$

*where* $\bar{x}=\sum_{i=1}^{n}p_{i}x_{i}$ *and* $\bar{y}=\sum_{j=1}^{m}q _{j}y_{j}$, *then for every* $f\in \mathcal{K}_{1}^{c}(a,B)$ *we have*

$$\begin{aligned} &\dfrac{A-\bar{x}}{A-a}f(a)+\dfrac{\bar{x}-a}{A-a}f(A)-\sum_{i=1}^{n}p _{i}f(x_{i})\le \dfrac{B-\bar{y}}{B-b}f(b)+\dfrac{\bar{y}-b}{B-b}f(B)- \sum_{j=1}^{m}q_{j}f(y_{j}). \end{aligned}$$

Our first result is a generalization of the result from [14] stated above, in which it is allowed for $p_{i}, q _{j}$ to also be negative, with the sum not equal to zero, but with supplementary demands on $p_{i},q_{j}$ and $x_{i},y_{j}$ given by using the Green function *G* defined in (1.8).

### Theorem 5.2

*Let* $[\alpha , \beta ]\subseteq \mathbb{R}$ *be an interval and* $c\in \langle \alpha ,\beta \rangle $. *Let* $x_{i}\in [a_{1},b_{1}] \subseteq [\alpha , c]$, $a_{1} \neq b_{1}$, $p_{i}\in \mathbb{R}$ ($i=1,\ldots,n$), *and* $y_{j}\in [a_{2},b_{2}]\subseteq [c,\beta ]$, $a_{2} \neq b_{2}$, $q_{j}\in \mathbb{R}$ ($j=1,\ldots,m$) *be such that* $P_{n}\neq 0$ *and* $Q_{m}\neq 0$ *and*

5.1 $$\begin{aligned} C&:=\frac{b_{1}-\bar{x}}{b_{1}-a_{1}}a_{1}^{2}+\frac{\bar{x}-a_{1}}{b _{1}-a_{1}}b_{1}^{2}- \frac{1}{P_{n}}\sum_{i=1}^{n} p_{i} x_{i}^{2} \\ &=\frac{b_{2}-\bar{y}}{b_{2}-a_{2}}a_{2}^{2}+\frac{\bar{y}-a_{2}}{b _{2}-a_{2}}b_{2}^{2}- \frac{1}{Q_{m}}\sum_{j=1}^{m} q_{j} y_{j}^{2}. \end{aligned}$$

*If for all* $s\in [\alpha ,c]$ *we have*

5.2 $$\begin{aligned} \frac{1}{P_{n}}\sum_{i=1}^{n} p_{i} G(x_{i},s) &\le \frac{b_{1}- \bar{x}}{b_{1}-a_{1}}G(a_{1},s)+ \frac{\bar{x}-a_{1}}{b_{1}-a_{1}}G(b _{1},s), \end{aligned}$$

*and for all* $s\in [c,\beta ]$ *we have*

5.3 $$\begin{aligned} \frac{1}{Q_{m}}\sum_{j=1}^{m} q_{j} G(y_{j},s) &\le \frac{b_{2}- \bar{y}}{b_{2}-a_{2}}G(a_{2},s)+ \frac{\bar{y}-a_{2}}{b_{2}-a_{2}}G(b _{2},s), \end{aligned}$$

*where* $\bar{x}=\frac{1}{P_{n}}\sum_{i=1}^{n} p_{i} x_{i}$, $\bar{y}=\frac{1}{Q _{m}}\sum_{j=1}^{m} q_{j} y_{j}$ *and the function* *G* *is defined in* (1.8), *then for every continuous function* $\varphi \in \mathcal{K}_{1}^{c}([\alpha ,\beta ])$ *we have*

5.4 $$\begin{aligned}& \frac{b_{1}-\bar{x}}{b_{1}-a_{1}} \varphi (a_{1})+\frac{\bar{x}-a_{1}}{b _{1}-a_{1}}\varphi (b_{1})-\frac{1}{P_{n}}\sum_{i=1}^{n} p_{i} \varphi (x_{i}) \\& \quad \le \frac{D}{2}C\le\frac{b_{2}-\bar{y}}{b_{2}-a_{2}}\varphi (a_{2})+\frac{\bar{y}-a _{2}}{b_{2}-a_{2}}\varphi (b_{2})-\frac{1}{Q_{m}}\sum_{j=1}^{m} q_{j} \varphi (y_{j}) . \end{aligned}$$

*The statement also holds if we reverse all signs of the inequalities in* (5.2), (5.3), *and* (5.4).

### Remark 5.3

If we set all $p_{i},q_{j}$ to be positive, then Theorem 5.2 becomes the result from [14] which is stated above in Theorem 5.1.

### Corollary 5.4

*Let the conditions from the previous theorem hold*.

(i) *If for all* $s\in [\alpha , c]$ *inequality* (5.2) *holds and for all* $s\in [c, \beta ]$ *inequality* (5.3) *holds*, *then for every continuous function* $\varphi \in \mathcal{K}_{2}^{c}([\alpha ,\beta ])$ *the reverse inequalities in* (5.4) *hold*.
(ii) *If for all* $s\in [\alpha , c]$ *the reversed inequality in* (5.2) *holds and for all* $s\in [c, \beta ]$ *the reversed inequality in* (5.3) *holds*, *then for every continuous function* $\varphi \in \mathcal{K}_{2}^{c}([\alpha ,\beta ])$ (5.4) *holds*.

### Remark 5.5

We can replace the equality from the condition (5.1) by a weaker condition in the analogous way as in Remark 4.5 from the previous chapter.

## The Hermite-Hadamard inequality

The classical Hermite-Hadamard inequality states that for a convex function $\varphi \colon [a,b]\to \mathbb{R}$ the following estimation holds:

6.1 $$\begin{aligned} \varphi \biggl( \frac{a+b}{2} \biggr) \leq \frac{1}{b-a} \int_{a}^{b} \varphi (x)\,dx\leq \frac{\varphi (a)+\varphi (b)}{2}. \end{aligned}$$

Its weighted form is proved by Fejér in [15]: If $\varphi \colon [a,b]\to \mathbb{R}$ is a convex function and $p\colon [a,b]\to \mathbb{R}$ nonnegative integrable function, symmetric with respect to the middle point $(a+b)/2$, then the following estimation holds:

6.2 $$\begin{aligned} \varphi \biggl( \frac{a+b}{2} \biggr) \int_{a}^{b} p(x)\,dx\leq \int_{a} ^{b} \varphi (x)p(x)\,dx\leq \frac{\varphi (a)+\varphi (b)}{2} \int _{a}^{b} p(x)\,dx. \end{aligned}$$

Fink in [16] discussed the generalization of (6.1) by separately looking the left and right side of the inequality and considering certain signed measures. In their paper [17], the authors gave a complete characterization of the right side of the Hermite-Hadamard inequality.

Rodić Lipanović, Pečarić, and Perić in [11] obtained the complete characterization for the left and the right side of the generalized Hermite-Hadamard inequality for the real Stieltjes measure.

In this section a Levinson type generalization of the Hermite-Hadamard inequality for signed measures will be given as a consequence of the results given in Sections 2 and 4.

Here we use the following notation:

$$\begin{aligned} \tilde{x}=\frac{\int_{a_{1}}^{b_{1}} x\,d\lambda (x)}{\int_{a_{1}}^{b_{1}}d\lambda (x)}\quad \mbox{and}\quad \tilde{y}= \frac{\int_{a_{2}}^{b_{2}} y\,d\mu (y)}{\int_{a_{2}}^{b_{2}}d\mu (y)}. \end{aligned}$$

### Corollary 6.1

*Let* $[\alpha , \beta ]\subseteq \mathbb{R}$ *be an interval and* $c\in \langle \alpha ,\beta \rangle $ *and let* $[a_{1},b_{1}]\subseteq [\alpha , c]$ *and* $[a_{2},b_{2}]\subseteq [c,\beta ]$. *Let* $\lambda \colon [a_{1},b_{1}]\to \mathbb{R}$ *and* $\mu \colon [a_{2},b_{2}] \to \mathbb{R}$ *be continuous functions or functions of bounded variation such that* $\lambda (a_{1})\neq \lambda (b_{1})$, $\mu (a _{2})\neq \mu (b_{2})$ *and* $\tilde{x}\in [\alpha ,c]$, $\tilde{y} \in [c,\beta ]$, *and such that*

6.3 $$\begin{aligned} C:=\frac{\int_{a_{1}}^{b_{1}} x^{2}\,d\lambda (x)}{\int_{a_{1}}^{b_{1}}d\lambda (x)}-\tilde{x}^{2}=\frac{\int_{a_{2}}^{b_{2}} y^{2}\,d\mu (y)}{ \int_{a_{2}}^{b_{2}}d\mu (y)}- \tilde{y}^{2} . \end{aligned}$$

*If for all* $s_{1}\in [\alpha ,c]$ *and for all* $s_{2}\in [c,\beta ]$ *the inequalities*

6.4 $$\begin{aligned} G(\tilde{x},s_{1})\le \frac{\int_{a_{1}}^{b_{1}} G(x,s_{1})\,d\lambda (x)}{ \int_{a_{1}}^{b_{1}}d\lambda (x)}\quad \textit{and} \quad G(\tilde{y},s_{2}) \le \frac{\int_{a_{2}}^{b_{2}} G(y,s_{2})\,d\mu (y)}{ \int_{a_{2}}^{b_{2}}d\mu (y)} \end{aligned}$$

*hold*, *where the function* *G* *is defined in* (1.8), *then for every continuous function* $\varphi \in \mathcal{K}_{1}^{c}([\alpha , \beta ])$ *we have*

6.5 $$\begin{aligned} \frac{\int_{a_{1}}^{b_{1}} \varphi (x)\,d\lambda (x)}{\int_{a_{1}}^{b _{1}}d\lambda (x)}-\varphi (\tilde{x})\le \frac{D}{2}C \le \frac{ \int_{a_{2}}^{b_{2}} \varphi (y)\,d\mu (y)}{\int_{a_{2}}^{b_{2}}d\mu (y)}- \varphi (\tilde{y}). \end{aligned}$$

*The statement also holds if we reverse all signs of the inequalities in* (6.4) *and* (6.5).

### Remark 6.2

Let the conditions from the previous corollary hold.

(i) If for all $s_{1}\in [\alpha , c]$ and $s_{2}\in [c, \beta ]$ inequalities (6.4) hold, then for every continuous function $\varphi \in \mathcal{K}_{2}^{c}([\alpha ,\beta ])$ the reverse inequalities in (6.5) hold.
(ii) If for all $s_{1}\in [\alpha , c]$ and $s_{2}\in [c, \beta ]$ the reversed inequalities in (6.4) hold, then for every continuous function $\varphi \in \mathcal{K}_{2}^{c}([ \alpha ,\beta ])$ (6.5) holds.

Note that for the Levinson type generalization of the left-side inequality of the generalized Hermite-Hadamard inequality it is necessary to demand that $\tilde{x}\in [\alpha , c]$ and $\tilde{y} \in [c,\beta ]$.

### Remark 6.3

If in Remark 2.3 we put $f(x)=x$ and $g(x)=x$, we can obtain weaker conditions instead of equality (6.3) under which inequality (6.5) holds.

Similarly, from the results given in the fourth section we get the Levinson type generalization of the right-side inequality of the generalized Hermite-Hadamard inequality. Here we allow that the mean value *x̃* goes outside of the interval $[\alpha , c]$ and *ỹ* outside of the interval $[c,\beta ]$.

### Corollary 6.4

*Let* $[\alpha , \beta ]\subseteq \mathbb{R}$ *be an interval and* $c\in \langle \alpha ,\beta \rangle $, *and let* $[a_{1},b_{1}]\subseteq [\alpha , c]$ *and* $[a_{2},b_{2}]\subseteq [c,\beta ]$. *Let* $\lambda \colon [a_{1},b_{1}]\to \mathbb{R}$ *and* $\mu \colon [a_{2},b_{2}] \to \mathbb{R}$ *be continuous functions or functions of bounded variation such that* $\lambda (a_{1})\neq \lambda (b_{1})$ *and* $\mu (a_{2})\neq \mu (b_{2})$ *and such that*

6.6 $$\begin{aligned} C&:=\frac{b_{1}-\tilde{x}}{b_{1}-a_{1}}a_{1}^{2}+\frac{\tilde{x}-a _{1}}{b_{1}-a_{1}}b_{1}^{2}- \frac{\int_{a_{1}}^{b_{1}} x^{2}\,d\lambda (x)}{ \int_{a_{1}}^{b_{1}}d\lambda (x)} \\ &=\frac{b_{2}-\tilde{y}}{b_{2}-a_{2}}a_{2}^{2}+\frac{\tilde{y}-a_{2}}{b _{2}-a_{2}}b_{2}^{2}- \frac{\int_{a_{2}}^{b_{2}} y^{2}\,d\mu (y)}{ \int_{a_{2}}^{b_{2}}d\mu (y)}. \end{aligned}$$

*If for all* $s\in [\alpha ,c]$ *we have*

6.7 $$\begin{aligned} \frac{\int_{a_{1}}^{b_{1}}G ( x,s )\,d\lambda (x)}{\int_{a_{1}} ^{b_{1}}d\lambda (x)} &\le \frac{b_{1}-\tilde{x}}{b_{1}-a_{1}}G(a_{1},s)+ \frac{ \tilde{x}-a_{1}}{b_{1}-a_{1}}G(b_{1},s), \end{aligned}$$

*and for all* $s\in [c,\beta ]$ *we have*

6.8 $$\begin{aligned} \frac{\int_{a_{2}}^{b_{2}}G ( y,s )\,d\mu (y)}{\int_{a_{2}} ^{b_{2}}d\mu (y)} &\le \frac{b_{2}-\tilde{y}}{b_{2}-a_{2}}G(a_{2},s)+ \frac{ \tilde{y}-a_{2}}{b_{2}-a_{2}}G(b_{2},s), \end{aligned}$$

*where the function* *G* *is defined in* (1.8), *then for every continuous function* $\varphi \in \mathcal{K}_{1}^{c}([\alpha ,\beta ])$ *we have*

6.9 $$\begin{aligned} &\frac{b_{1}-\tilde{x}}{b_{1}-a_{1}} \varphi (a_{1})+\frac{\tilde{x}-a _{1}}{b_{1}-a_{1}}\varphi (b_{1})-\frac{\int_{a_{1}}^{b_{1}} \varphi ( x )\,d\lambda (x)}{\int_{a_{1}}^{b_{1}}d\lambda (x)} \\ &\quad \le \frac{D}{2}C\le\frac{b_{2}-\tilde{y}}{b_{2}-a_{2}}\varphi (a_{2})+ \frac{\tilde{y}-a _{2}}{b_{2}-a_{2}}\varphi (b_{2})-\frac{\int_{a_{2}}^{b_{2}}\varphi ( y )\,d\mu (y)}{\int_{a_{2}}^{b_{2}}d\mu (y)}. \end{aligned}$$

*The statement also holds if we reverse all signs of the inequalities in* (6.7), (6.8) *and* (6.9).

### Remark 6.5

Let the conditions from the previous theorem hold.

(i) If for all $s\in [\alpha , c]$ inequality (6.7) holds and for all $s\in [c,\beta ]$ inequality (6.8) holds, then for every continuous function $\varphi \in \mathcal{K}_{2}^{c}([\alpha ,\beta ])$ the reverse inequalities in (6.9) hold.
(ii) If for all $s\in [\alpha , c]$ the reversed inequality in (6.7) holds and for all $s\in [c,\beta ]$ the reversed inequality in (6.8) holds, then for every continuous function $\varphi \in \mathcal{K}_{2}^{c}([\alpha ,\beta ])$ (6.9) holds.

### Remark 6.6

If in Remark 4.5 we put $f(x)=x$ and $g(x)=x$, we can obtain analogous weaker conditions instead of equality (6.6) under which inequality (6.9) holds.

It is easy to see that for $\lambda (x)=x$ and $\mu (x)=x$ the conditions (6.4), (6.7) and (6.8) are always fulfilled. In that way we can obtain a Levinson type generalization of both sides in the classical weighted Hermite-Hadamard inequality.

### Corollary 6.7

*Let* $[\alpha , \beta ]\subseteq \mathbb{R}$ *be an interval and* $c\in \langle \alpha ,\beta \rangle $, *and let* $[a_{1},b_{1}]\subseteq [\alpha , c]$ *and* $[a_{2},b_{2}]\subseteq [c,\beta ]$.

(i) *If* $C:=\frac{1}{12}(b_{2}-a_{2})^{2}=\frac{1}{12}(b_{1}-a _{1})^{2}$ *holds*, *then for every continuous function* $\varphi \in \mathcal{K}_{1}^{c}([\alpha ,\beta ])$
  $$\begin{aligned}& \frac{1}{b_{1}-a_{1}} \int_{a_{1}}^{b_{1}} \varphi (x)\,dx-\varphi \biggl(\dfrac{a_{1}+b_{1}}{2} \biggr) \\& \quad \le \frac{D}{2}C \le \frac{1}{b_{2}-a_{2}} \int_{a_{2}}^{b_{2}} \varphi (x)\,dx- \varphi \biggl( \dfrac{a_{2}+b_{2}}{2} \biggr) . \end{aligned}$$
(ii) *If* $C:=\frac{1}{6}(b_{2}-a_{2})^{2}=\frac{1}{6}(b_{1}-a _{1})^{2}$ *holds*, *then for every continuous function* $\varphi \in \mathcal{K}_{1}^{c}([\alpha ,\beta ])$
  $$\begin{aligned}& \frac{\varphi (a_{1})+\varphi (b_{1})}{2} -\frac{1}{b_{1}-a_{1}} \int_{a_{1}}^{b_{1}} \varphi ( x )\,dx \\& \quad \le\frac{D}{2}C\le\frac{\varphi (a_{2})+\varphi (b_{2})}{2}-\frac{1}{b_{2}-a_{2}} \int_{a_{2}}^{b_{2}} \varphi ( x )\,dx . \end{aligned}$$

*If* $\varphi \in \mathcal{K}_{2}^{c}([\alpha ,\beta ])$, *then the inequalities in* (i) *and* (ii) *are reversed*.

## The inequalities of Giaccardi and Petrović

The well-known Petrović inequality [18] for convex function $f \colon [0, a] \to \mathbb{R}$ is given by

7.1 $$ \sum_{i=1}^{n} f(x_{i}) \le f \Biggl(\sum_{i=1}^{n} x_{i} \Biggr)+(n-1)f(0), $$

where $x_{i}$ $(i=1,\ldots,n)$ are nonnegative numbers such that $x_{1},\ldots,x_{n}$, $\sum_{i=1}^{n} x_{i} \in [0,a]$.

The following generalization of (7.1) is given by Giaccardi (see [3] and [19]).

### Theorem 7.1

Giaccardi, [19]

*Let* $\mathbf{p}=(p _{1},\ldots, p_{n})$ *be a nonnegative* *n*-*tuple and* $\mathbf{x}=(x_{1},\ldots, x_{n})$ *be a real* *n*-*tuple such that*

$$\begin{aligned}& (x_{i}-x_{0}) \Biggl( \sum_{j=1}^{n}p_{j}x_{j}-x_{i} \Biggr) \ge 0, \quad i=1,\ldots,n, \\& x_{0}, \qquad \sum_{i=1}^{n} p_{i}x_{i} \in [a,b] \quad \textit{and} \quad \sum_{i=1}^{n}p_{i}x_{i} \neq x_{0}. \end{aligned}$$

*If* $f \colon [a,b] \to \mathbb{R}$ *is a convex function*, *then*

7.2 $$ \sum_{i=1}^{n} p_{i}f(x_{i}) \le Af \Biggl( \sum _{i=1}^{n} p_{i}x_{i} \Biggr)+B \Biggl( \sum_{i=1}^{n} p_{i}-1 \Biggr) f(x_{0}), $$

*where*

$$ A=\dfrac{\sum_{i=1}^{n} p_{i}(x_{i}-x_{0})}{\sum_{i=1}^{n} p_{i}x_{i}-x _{0}}, \qquad B=\dfrac{\sum_{i=1}^{n} p_{i}x_{i}}{\sum_{i=1}^{n} p_{i}x _{i}-x_{0}}. $$

In this section we will use an analogous technique as in the previous sections to obtain a Levinson type generalization of the Giaccardi inequality for *n*-tuples **p** of real numbers which are not necessarily nonnegative. As a simple consequence, we will obtain a Levinson type generalization of the original Giaccardi inequality (7.2). In order to do so, we first need to state two results from [11].

### Theorem 7.2

[11]

*Let* $x_{i} \in [a,b]\subseteq [\alpha ,\beta ]$, $a \neq b$, $p_{i} \in \mathbb{R}$ ($i=1,\ldots,n$) *be such that* $P_{n}\neq 0$. *Then the following two statements are equivalent*:

(1): *For every continuous convex function* $f: [\alpha , \beta ]\rightarrow \mathbb{R}$
  7.3 $$ \frac{1}{P_{n}}\sum_{i=1}^{n} p_{i} f(x_{i}) \leq \frac{b-\bar{x}}{b-a}f(a)+ \frac{\bar{x}-a}{b-a}f(b) $$
  *holds*.
(2): *For all* $s\in [\alpha , \beta ]$
  7.4 $$ \frac{1}{P_{n}}\sum_{i=1}^{n} p_{i} G(x_{i},s)\leq \frac{b-\bar{x}}{b-a}G(a,s)+ \frac{\bar{x}-a}{b-a}G(b,s) $$
  *holds*, *where the function* $G: [\alpha , \beta ]\times [\alpha , \beta ] \rightarrow \mathbb{R}$ *is defined in* (1.8).

*Moreover*, *the statements* (1) *and* (2) *are also equivalent if we change the sign of the inequality in both inequalities*, *in* (7.3) *and in* (7.4).

### Corollary 7.3

[11]

*Under the conditions from the previous theorem*, *the following two statements are also equivalent*:

(1′): *For every continuous concave function* $\varphi : [ \alpha , \beta ]\rightarrow \mathbb{R}$ *the reverse inequality in* (7.3) *holds*.
(2′): *For all* $s\in [\alpha , \beta ]$ *inequality* (7.4) *holds*.

*Moreover*, *the statements* (1′) *and* (2′) *are also equivalent if we change the sign of inequality in both statements* (1′) *and* (2′).

Our first result is a Levinson type generalization of the Giaccardi inequality for *n*-tuples **p** and *m*-tuples **q** of arbitrary real numbers instead of nonnegative real numbers.

### Theorem 7.4

*Let* $[\alpha , \beta ]\subseteq \mathbb{R}$ *be an interval and* $c\in \langle \alpha ,\beta \rangle $ *such that* $[a_{1},b_{1}]\subseteq [\alpha , c]$ *and* $[a_{2},b_{2}]\subseteq [c,\beta ]$. *Let* **p** *and* **x** *be* *n*-*tuples of real numbers*, *and let* **q** *and* **y** *be* *m*-*tuples of real numbers such that* $P_{n}=\sum_{i=1} ^{n}p_{i}\neq 0$, $Q_{m}=\sum_{i=1}^{m}q_{i}\neq 0$, *and*

7.5 $$ \begin{aligned} &(x_{j}-x_{0}) \Biggl( \sum_{i=1}^{n}p_{i}x_{i}-x_{j} \Biggr) \ge 0 \quad (j=1,\ldots,n); \\ &x_{0}, \qquad \sum_{i=1}^{n} p_{i}x_{i} \in [a_{1},b_{1}]; \qquad \sum_{i=1}^{n}p_{i}x_{i} \neq x_{0}; \\ &(y_{j}-y_{0}) \Biggl( \sum_{i=1}^{m}q_{i}y_{i}-y_{j} \Biggr) \ge 0 \quad (j=1,\ldots,m); \\ &y_{0}, \qquad \sum_{i=1}^{m} q_{i}y_{i} \in [a_{2},b_{2}]; \qquad \sum_{i=1}^{m}q_{i}y_{i} \neq y_{0}. \end{aligned} $$

*Let*

7.6 $$\begin{aligned} C&:=A_{1} \Biggl( \sum_{i=1}^{n}p_{i}x_{i} \Biggr) ^{2}+B_{1} \Biggl( \sum _{i=1}^{n}p_{i}-1 \Biggr) x_{0}^{2}-\sum_{i=1}^{n}p_{i}x_{i}^{2} \\ &=A_{2} \Biggl( \sum_{j=1}^{m}q_{j}y_{j} \Biggr) ^{2}+B_{2} \Biggl( \sum_{j=1} ^{m}q_{j}-1 \Biggr) y_{0}^{2}-\sum _{j=1}^{m}q_{j}y_{j}^{2}, \end{aligned}$$

*where*

7.7 $$ \begin{aligned} A_{1} =\dfrac{\sum_{i=1}^{n} p_{i}(x_{i}-x_{0})}{\sum_{i=1}^{n} p_{i}x _{i}-x_{0}}, \qquad B_{1}=\dfrac{\sum_{i=1}^{n} p_{i}x_{i}}{\sum_{i=1}^{n} p_{i}x_{i}-x_{0}}, \\ A_{2} =\dfrac{\sum_{j=1}^{m} q_{j}(y_{j}-y_{0})}{\sum_{j=1}^{m} q _{j}y_{j}-y_{0}}, \qquad B_{2}=\dfrac{\sum_{j=1}^{m}q_{j}y_{j}}{\sum_{j=1} ^{m}q_{j}y_{j}-y_{0}}. \end{aligned} $$

*If* $P_{n}\cdot Q_{m}>0$ *and for all* $s\in [\alpha ,\beta ]$ *and the function* *G* *defined in* (1.8) *the inequalities*

7.8 $$\begin{aligned}& \sum_{i=1}^{n} p_{i} G(x_{i},s) \le A_{1}G \Biggl( \sum _{i=1}^{n}p_{i}x _{i},s \Biggr) +B_{1} \Biggl( \sum_{i=1}^{n}p_{i}-1 \Biggr) G(x_{0},s), \end{aligned}$$

7.9 $$\begin{aligned}& \sum_{j=1}^{m} q_{j} G(y_{j},s) \le A_{2}G \Biggl( \sum _{j=1}^{m}q_{j}y _{j},s \Biggr) +B_{2} \Biggl( \sum_{j=1}^{m}q_{j}-1 \Biggr) G(y_{0},s), \end{aligned}$$

*hold*, *then for every continuous function* $\varphi \in \mathcal{K}_{1} ^{c}([\alpha ,\beta ])$ *we have*

7.10 $$\begin{aligned}& A_{1}\varphi \Biggl( \sum_{i=1}^{n}p_{i}x_{i} \Biggr)+ B_{1} \Biggl( \sum_{i=1}^{n} p_{i}-1 \Biggr) \varphi(x_{0})-\sum_{i=1}^{n}p_{i}\varphi (x_{i}) \\& \quad \le \frac{D}{2}C\le A_{2}\varphi \Biggl( \sum_{j=1}^{m} q_{j}y_{j} \Biggr)+B_{2} \Biggl( \sum _{j=1}^{m} q_{j}-1 \Biggr) \varphi (y_{0})-\sum_{j=1}^{m} q_{j} \varphi (y_{j}) . \end{aligned}$$

*The statement also holds if we reverse all signs of the inequalities in* (7.8), (7.9), *and* (7.10).

### Proof

We follow the same idea as in the proof of Theorem 4.3 from Section 4. We apply Theorem 7.2 and Corollary 7.3 to the function $\phi (x)=\varphi (x)-\frac{D}{2}x ^{2}$, which is concave on $[\alpha ,c]$ and convex on $[c,\beta ]$. We set $a=\min \{x_{0}, \sum_{i=1}^{n}p_{i}x_{i}\}$, $b=\max \{x_{0}, \sum_{i=1}^{n}p_{i}x_{i}\}$ on $[\alpha , c]$, and then we set $a=\min \{y_{0}, \sum_{j=1}^{m}q_{j}y_{j}\}$ and $b=\max \{y_{0}, \sum_{j=1}^{m}q_{j}y_{j}\}$ on $[c, \beta ]$, as well as consider the signs of $P_{n}$ and $Q_{m}$. We omit the details. □

### Corollary 7.5

*Let the assumptions from the previous theorem hold*.

(i) *If* $P_{n}>0$ *and* $Q_{m}<0$ *and if for all* $s\in [\alpha , \beta ]$ *inequality* (7.8) *holds and inequality* (7.9) *is reversed*, *then* (7.10) *holds*.
(ii) *If* $P_{n}<0$ *and* $Q_{m}>0$ *and if for all* $s\in [\alpha , \beta ]$ *inequality* (7.8) *is reversed and inequality* (7.9) *holds*, *then* (7.10) *holds*.

*Statements* (i) *and* (ii) *also hold if we reverse the signs in all of the inequalities*.

### Corollary 7.6

*Let the assumptions from the previous theorem hold*.

(i) *If* $P_{n}\cdot Q_{m}>0$ *and if for all* $s\in [\alpha , \beta ]$ *inequalities* (7.8) *and* (7.9) *hold*, *then for every continuous function* $\varphi \in \mathcal{K}_{2} ^{c}([\alpha ,\beta ])$ *the reversed inequality in* (7.10) *holds*.
(ii) *If* $P_{n}\cdot Q_{m}>0$ *and if for all* $s\in [\alpha , \beta ]$ *inequalities* (7.8) *and* (7.9) *are reversed*, *then for every continuous function* $\varphi \in \mathcal{K}_{2}^{c}([\alpha ,\beta ])$ (7.10) *holds*.
(iii) *If* $P_{n}>0$ *and* $Q_{m}<0$ *and if for all* $s\in [\alpha ,\beta ]$ *inequality* (7.8) *holds and inequality* (7.9) *is reversed*, *then for every continuous function* $\varphi \in \mathcal{K}_{2}^{c}([\alpha ,\beta ])$ *the reversed inequality in* (7.10) *holds*.
(iv) *If* $P_{n}<0$ *and* $Q_{m}>0$ *and if for all* $s\in [\alpha , \beta ]$ *inequality* (7.8) *is reversed and inequality* (7.9) *holds*, *then for every continuous function* $\varphi \in \mathcal{K}_{2}^{c}([\alpha ,\beta ])$ *the reversed inequality in* (7.10) *holds*.

*Statements* (iii) *and* (iv) *also hold if we reverse the signs in all of the mentioned inequalities*.

### Remark 7.7

One needs to notice that if we set $p_{i}$ ($i=1,\ldots,n$) and $q_{j}$ ($j=1,\ldots,m$) to be positive, Theorem 7.4 becomes the Levinson type generalization of the original Giaccardi inequality (7.2).

### Remark 7.8

As in the previous sections, we can replace the equality (7.6) by a weaker condition

7.11 $$\begin{aligned} C_{1}&:= \frac{D}{2} \Biggl[ A_{1} \Biggl( \sum _{i=1}^{n}p_{i}x_{i} \Biggr) ^{2}+B_{1} \Biggl( \sum_{i=1}^{n}p_{i}-1 \Biggr) x_{0}^{2}-\sum_{i=1}^{n}p _{i}x_{i}^{2} \Biggr] \\ &\le C_{2}:=\frac{D}{2} \Biggl[A_{2} \Biggl( \sum _{j=1}^{m}q_{j}y_{j} \Biggr) ^{2}+B_{2} \Biggl( \sum_{j=1}^{m}q_{j}-1 \Biggr) y_{0}^{2}-\sum_{j=1}^{m}q _{j}y_{j}^{2} \Biggr], \end{aligned}$$

and then (7.10) becomes

$$\begin{aligned}& A_{1}\varphi \Biggl( \sum_{i=1}^{n} p_{i}x_{i} \Biggr)+ B_{1} \Biggl( \sum _{i=1}^{n} p_{i}-1 \Biggr) \varphi (x_{0})-\sum_{i=1}^{n} p_{i}\varphi (x_{i}) \le C_{1} \\& \quad \le C_{2}\le A_{2}\varphi \Biggl( \sum _{j=1}^{m} q_{j}y_{j} \Biggr)+B _{2} \Biggl( \sum_{j=1}^{m} q_{j}-1 \Biggr) \varphi (y_{0})-\sum _{j=1} ^{m} q_{j}\varphi (y_{j}) . \end{aligned}$$

Since $\varphi_{-}''(c)\le D\le \varphi_{+}''(c)$ (see [5]), if additionally *φ* is convex (resp. concave), the condition (7.11) can be further weakened to

$$\begin{aligned}& A_{1} \Biggl( \sum_{i=1}^{n}p_{i}x_{i} \Biggr) ^{2}+ B_{1} \Biggl( \sum _{i=1} ^{n}p_{i}-1 \Biggr) x_{0}^{2}-\sum_{i=1}^{n}p_{i}x_{i}^{2} \\& \quad \le A_{2} \Biggl( \sum_{j=1}^{m}q_{j}y_{j} \Biggr) ^{2}+B_{2} \Biggl( \sum_{j=1}^{m}q_{j}-1 \Biggr) y_{0}^{2}-\sum_{j=1}^{m}q_{j}y_{j}^{2}. \end{aligned}$$

## Mean-value theorems

Let $f\colon [a_{1},b_{1}]\to \mathbb{R}$ and $g\colon [a_{2},b_{2}] \to \mathbb{R}$ be continuous functions, $[\alpha , \beta ]\subseteq \mathbb{R}$ an interval and $c\in \langle \alpha ,\beta \rangle $ such that $f([a_{1},b_{1}])=[m_{1},M_{1}]\subseteq [\alpha , c]$ and $g([a_{2},b_{2}])=[m_{2},M_{2}]\subseteq [c,\beta ]$. Let $\lambda \colon [a_{1},b_{1}]\to \mathbb{R}$ and $\mu \colon [a_{2},b_{2}] \to \mathbb{R}$ be continuous functions or functions of bounded variation such that $\lambda (a_{1})\neq \lambda (b_{1})$ and $\mu (a_{2})\neq \mu (b_{2})$, and let $\varphi \in \mathcal{K}_{1} ^{c}([\alpha ,\beta ])$ be a continuous function.

Motivated by the results obtained in previous sections, we define the following linear functionals which, respectively, represent the difference between the right and the left side of inequalities (2.3) and (4.7):

8.1 $$\begin{aligned} \Gamma_{J} (\varphi ) &=\frac{\int_{a_{2}}^{b_{2}} \varphi (g(x))\,d\mu (x)}{\int_{a_{2}}^{b_{2}}d\mu (x)}- \varphi (\bar{g}) - \frac{ \int_{a_{1}}^{b_{1}} \varphi (f(x))\,d\lambda (x)}{\int_{a_{1}}^{b_{1}}d\lambda (x)}+\varphi (\bar{f}) , \end{aligned}$$

where $\bar{f} \in [\alpha , c]$, $\bar{g} \in [c,\beta ]$;

8.2 $$\begin{aligned} \Gamma_{\mathrm{ELR}}(\varphi ) ={}&\frac{M_{2}-\bar{g}}{M_{2}-m_{2}}\varphi (m _{2})+ \frac{\overline{g}-m_{2}}{M_{2}-m_{2}}\varphi (M_{2})-\frac{ \int_{a_{2}}^{b_{2}}\varphi ( g(x) )\,d\mu (x)}{\int_{a_{2}} ^{b_{2}}d\mu (x)} \\ & {}-\frac{M_{1}-\bar{f}}{M_{1}-m_{1}}\varphi (m_{1})-\frac{\bar{f}-m_{1}}{M _{1}-m_{1}}\varphi (M_{1})+\frac{\int_{a_{1}}^{b_{1}} \varphi ( f(x) )\,d\lambda (x)}{\int_{a_{1}}^{b_{1}}d\lambda (x)}, \end{aligned}$$

where $m_{1}\neq M_{1}$ and $m_{2}\neq M_{2}$.

We have:

(i) $\Gamma_{J} (\varphi )\ge 0$, when (2.1) holds and for all $s_{1}\in [\alpha ,c]$, $s_{2}\in [c,\beta ]$ (2.2) holds;
(ii) $\Gamma_{\mathrm{ELR}} (\varphi )\ge 0$, when (4.4) holds, and for all $s\in [\alpha ,c]$ (4.5) holds and for all $s\in [c,\beta ]$ (4.6) holds.

In the following two theorems we give the mean-value theorems of the Lagrange and Cauchy type, respectively.

### Theorem 8.1

*Let* $f\colon [a_{1},b_{1}]\to \mathbb{R}$ *and* $g\colon [a_{2},b_{2}] \to \mathbb{R}$ *be continuous functions*, $[\alpha , \beta ]\subseteq \mathbb{R}$ *an interval and* $c\in \langle \alpha ,\beta \rangle $ *such that* $f([a_{1},b_{1}])=[m_{1},M_{1}]\subseteq [\alpha , c]$ *and* $g([a_{2},b_{2}])=[m_{2},M_{2}]\subseteq [c,\beta ]$. *Let* $\lambda \colon [a_{1},b_{1}]\to \mathbb{R}$ *and* $\mu \colon [a_{2},b_{2}] \to \mathbb{R}$ *be continuous functions or functions of bounded variation such that* $\lambda (a_{1})\neq \lambda (b_{1})$, $\mu (a _{2})\neq \mu (b_{2})$. *Let* $\Gamma_{J}$ *and* $\Gamma_{\mathrm{ELR}}$ *be linear functionals defined above*, *and let* $\varphi \in C^{3}([\alpha ,\beta ])$.

(i) *If* (2.1) *holds and for all* $s_{1}\in [ \alpha ,c]$, $s_{2}\in [c,\beta ]$ (2.2) *holds*, *then there exists* $\xi_{1} \in [\alpha ,\beta ]$ *such that*
  8.3 $$\begin{aligned} \Gamma_{J} (\varphi )=\dfrac{\varphi '''(\xi_{1})}{6} \biggl[\frac{ \int_{a_{2}}^{b_{2}} g^{3}(x)\,d\mu (x)}{\int_{a_{2}}^{b_{2}}d\mu (x)}- \frac{ \int_{a_{1}}^{b_{1}}f^{3}(x)\,d\lambda (x)}{\int_{a_{1}}^{b_{1}}d\lambda (x)}+\bar{f}^{3}- \bar{g}^{3} \biggr]. \end{aligned}$$
(ii) *If* (4.4) *holds*, *and for all* $s\in [ \alpha ,c]$ (4.5) *holds and for all* $s\in [c, \beta ]$ (4.6) *holds*, *then there exists* $\xi_{2} \in [\alpha ,\beta ]$ *such that*
  8.4 $$\begin{aligned} \Gamma_{\mathrm{ELR}} (\varphi ) ={}&\dfrac{\varphi '''(\xi_{2})}{6} \biggl[ \frac{M _{2}-\bar{g}}{M_{2}-m_{2}}m_{2}^{3}+\frac{\overline{g}-m_{2}}{M_{2}-m _{2}}M_{2}^{3}- \frac{\int_{a_{2}}^{b_{2}} g^{3}(x)\,d\mu (x)}{\int_{a _{2}}^{b_{2}}d\mu (x)} \\ &{}-\frac{M_{1}-\bar{f}}{M_{1}-m_{1}}m_{1}^{3}-\frac{\bar{f}-m_{1}}{M _{1}-m_{1}}M_{1}^{3}+ \frac{\int_{a_{1}}^{b_{1}} f^{3}(x)\,d\lambda (x)}{ \int_{a_{1}}^{b_{1}}d\lambda (x)} \biggr]. \end{aligned}$$

### Proof

Since $\varphi '''(x)$ is continuous on $[\alpha , \beta ]$, it attains its minimum and maximum value on $[\alpha , \beta ]$, *i.e.* there exist $m=\min_{x\in [\alpha ,\beta ]} \varphi '''(x)$ and $M=\max_{x\in [\alpha ,\beta ]}\varphi '''(x)$. The functions $\varphi_{1}, \varphi_{2}: [\alpha ,\beta ]\to \mathbb{R}$ defined by

$$\begin{aligned} \varphi_{1}(x)=\varphi (x)-\dfrac{m}{6}x^{3} \quad \mbox{and} \quad \varphi_{2}(x)=\dfrac{M}{6}x^{3}- \varphi (x) \end{aligned}$$

are 3-convex because $\varphi_{1}'''(x)\ge 0$ and $\varphi_{2}'''(x) \ge 0$, so from Remark 1.6 it follows that they belong to the class $\mathcal{K}_{1}^{c}([\alpha ,\beta ])$. From Theorem 2.1 it follows that $\Gamma_{J} (\varphi_{1})\ge 0$ and $\Gamma_{J} (\varphi_{2})\ge 0$, and from Theorem 4.3 it follows that $\Gamma_{\mathrm{ELR}} (\varphi_{1})\ge 0$ and $\Gamma_{\mathrm{ELR}} (\varphi_{2}) \ge 0$ and so we get

8.5 $$\begin{aligned}& \dfrac{m}{6}\Gamma_{J} (\tilde{\varphi })\le \Gamma_{J} (\varphi )\le \dfrac{M}{6}\Gamma_{J} ( \tilde{\varphi }), \end{aligned}$$

8.6 $$\begin{aligned}& \dfrac{m}{6}\Gamma_{\mathrm{ELR}} (\tilde{\varphi })\le \Gamma_{\mathrm{ELR}} (\varphi)\le \dfrac{M}{6}\Gamma_{\mathrm{ELR}} ( \tilde{\varphi }), \end{aligned}$$

where $\tilde{\varphi }(x)=x^{3}$. Since the function *φ̃* is 3-convex, we have $\tilde{\varphi } \in \mathcal{K}_{1}^{c}([\alpha ,\beta ])$, so by applying Theorem 2.1 (resp. Theorem 4.3) we get $\Gamma_{J} ( \tilde{\varphi })\ge 0$ (resp. $\Gamma_{\mathrm{ELR}} (\tilde{\varphi })\ge 0$). If $\Gamma_{J} (\tilde{\varphi })=0$ (resp. $\Gamma_{\mathrm{ELR}} ( \tilde{\varphi })=0$), then (8.5) implies $\Gamma_{J} ( \varphi )=0$ (resp. (8.6) implies $\Gamma_{\mathrm{ELR}} (\varphi )=0$), so (8.3) (resp. (8.4)) holds for every $\xi \in [\alpha ,\beta ]$. Otherwise, dividing (8.5) by $\Gamma_{J} (\tilde{\varphi })>0$ (resp. (8.6) by $\Gamma_{\mathrm{ELR}} (\tilde{\varphi })>0$) we get

$$\begin{aligned} \frac{m}{6}\le \dfrac{\Gamma_{J} (\varphi )}{\Gamma_{J} ( \tilde{\varphi })}\le \frac{M}{6} \quad \biggl( \mbox{resp. } \frac{m}{6}\le \dfrac{\Gamma_{\mathrm{ELR}} (\varphi )}{\Gamma_{\mathrm{ELR}} (\tilde{\varphi })}\le \frac{M}{6} \biggr), \end{aligned}$$

and continuity of $\varphi '''$ ensures the existence of $\xi_{1} \in [\alpha ,\beta ]$ satisfying (8.3) (resp. $\xi_{2} \in [\alpha ,\beta ]$ satisfying (8.4)). □

### Theorem 8.2

*Let the conditions from Theorem* 8.1 *hold*. *Let* $\varphi , \psi \in C^{3}([\alpha ,\beta ])$. *If* $\Gamma_{J} (\psi ) \neq 0$ *and* $\Gamma_{\mathrm{ELR}} (\psi )\neq 0$, *then there exist* $\xi_{1}, \xi_{2} \in [\alpha ,\beta ]$ *such that*

$$\begin{aligned} \dfrac{\Gamma_{J} (\varphi )}{\Gamma_{J} (\psi )}=\dfrac{\varphi '''( \xi_{1})}{\psi '''(\xi_{1})}\quad \textit{or}\quad \varphi '''(\xi_{1})=\psi '''(\xi_{1})=0 \end{aligned}$$

*and*

$$\begin{aligned} \dfrac{\Gamma_{\mathrm{ELR}} (\varphi )}{\Gamma_{\mathrm{ELR}} (\psi )}=\dfrac{\varphi '''(\xi_{2})}{\psi '''(\xi_{2})}\quad \textit{or}\quad \varphi '''(\xi_{2})=\psi '''(\xi_{2})=0. \end{aligned}$$

### Proof

Let us define a function $\chi \in C^{3}([\alpha , \beta ])$ by $\chi (x)=\Gamma_{J} (\psi )\varphi (x)-\Gamma_{J} ( \varphi )\psi (x)$. Due to the linearity of $\Gamma_{J} $ we have $\Gamma_{J} (\chi )=0$. Theorem 8.1 implies that there exist $\xi_{1}, \xi \in [\alpha ,\beta ]$ such that

$$\begin{aligned}& \Gamma_{J} (\chi )=\dfrac{\chi '''(\xi_{1})}{6}\Gamma_{J} ( \tilde{ \varphi }), \\& \Gamma_{J} (\psi )=\dfrac{\psi '''(\xi )}{6}\Gamma_{J} ( \tilde{ \varphi }), \end{aligned}$$

where $\tilde{\varphi }(x)=x^{3}$. Now we have $\Gamma_{J} ( \tilde{\varphi })\neq 0$, because otherwise we would have $\Gamma_{J} (\psi )=0$, which is a contradiction with the assumption $\Gamma_{J} ( \psi )\neq 0$. So we have

$$\begin{aligned} \chi '''(\xi_{1})= \Gamma_{J} (\psi )\varphi '''( \xi_{1})-\Gamma_{J} ( \varphi )\psi '''( \xi_{1})=0, \end{aligned}$$

and this gives us the first claim of the theorem. The second claim is proved in an analogous manner, by observing the linear functional $\Gamma_{\mathrm{ELR}}$ instead of $\Gamma_{J}$. □

### Remark 8.3

Note that if in Theorem 8.2 we set the function *ψ* to be $\psi (x)=x^{3}$, we get exactly Theorem 8.1.

### Remark 8.4

Note that if we set the functions *f*, *g*, *λ*, and *μ* from our theorems to fulfill the conditions from Jensen’s integral inequality or Jensen-Steffensen’s, or Jensen-Brunk’s, or Jensen-Boas’ inequality, then - applying that inequality on the function *G* which is continuous and convex in both variables - we see that in these cases for all $s_{1}\in [\alpha ,c]$, $s_{2}\in [c,\beta ]$ inequalities in (2.2) hold, and so from our results we directly get the results from the paper [10].

### Remark 8.5

If in the definition of the functional $\Gamma_{J}$ (resp. $\Gamma _{\mathrm{ELR}}$) we set $f(x)=x$ and $g(x)=x$, then we get a functional that represents the difference between the right and the left side of the left-hand part (resp. right-hand part) of the generalized Hermite-Hadamard inequality. In the same manner, adequate results of Lagrange and Cauchy type for those functionals can be derived directly from Theorem 8.1 and Theorem 8.2.

### Discrete case

Let $[\alpha , \beta ] \subseteq \mathbb{R}$ and $c\in \langle \alpha ,\beta \rangle $. Let $x_{i}\in [a_{1},b_{1}]\subseteq [\alpha ,c]$, $p_{i}\in \mathbb{R}$ ($i=1,\ldots,n$) be such that $P_{n}\neq 0$, and let $y_{j}\in [a_{2},b_{2}]\subseteq [c, \beta ]$, $q_{j}\in \mathbb{R}$ ($j=1,\ldots,m$) be such that $Q_{m}\neq 0$. Let $\varphi \in \mathcal{K}_{1}^{c}([\alpha ,\beta ])$ be a continuous function.

As before, motivated by the discrete results obtained in previous sections, we define the following linear functionals which, respectively, represent the difference between the right and the left side of inequalities (3.3), (5.4), and (7.10):

8.7 $$\begin{aligned} \Gamma_{J_{D}} (\varphi ) &=\frac{1}{Q_{m}}\sum _{j=1}^{m}q_{j}\varphi (y _{j})-\frac{1}{P_{n}}\sum_{i=1}^{n}p_{i} \varphi (x_{i})+\varphi ( \bar{x})-\varphi (\bar{y}) , \end{aligned}$$

where $\bar{x} \in [\alpha , c]$, $\bar{y} \in [c,\beta ]$;

8.8 $$\begin{aligned} \Gamma_{ELR_{D}} (\varphi ) ={}&\frac{b_{2}-\bar{y}}{b_{2}-a_{2}}\varphi (a_{2})+ \frac{\bar{y}-a_{2}}{b_{2}-a_{2}}\varphi (b_{2})-\frac{1}{Q _{m}}\sum _{j=1}^{m} q_{j} \varphi (y_{j}) \\ & {}-\frac{b_{1}-\bar{x}}{b_{1}-a_{1}}\varphi (a_{1})-\frac{\bar{x}-a_{1}}{b _{1}-a_{1}}\varphi (b_{1})+\frac{1}{P_{n}}\sum_{i=1}^{n} p_{i}\varphi ( x_{i}) , \end{aligned}$$

where $a_{1}\neq b_{1}$ and $a_{2}\neq b_{2}$;

8.9 $$\begin{aligned} \Gamma_{G} (\varphi ) ={}& A_{2}\varphi \Biggl( \sum _{j=1}^{m} q_{j}y_{j} \Biggr)+B_{2} \Biggl( \sum_{j=1}^{m} q_{j}-1 \Biggr) \varphi (y_{0})-\sum _{j=1}^{m} q_{j}\varphi (y_{j}) \\ & {}- A_{1}\varphi \Biggl( \sum_{i=1}^{n} p_{i}x_{i} \Biggr)-B_{1} \Biggl( \sum _{i=1}^{n} p_{i}-1 \Biggr) \varphi (x_{0})+\sum_{i=1}^{n} p_{i} \varphi (x_{i}), \end{aligned}$$

where the conditions (7.5) hold and $A_{1},B_{1},A_{2},B_{2}$ are defined in (7.7).

We have:

(i) $\Gamma_{J_{D}} (\varphi )\ge 0$, when (3.1) holds and for all $s_{1}\in [\alpha ,c]$, $s_{2}\in [c,\beta ]$ (3.2) holds;
(ii) $\Gamma_{ELR_{D}} (\varphi )\ge 0$, when (5.1) holds, and for all $s\in [\alpha ,c]$ (5.2) holds and for all $s\in [c,\beta ]$ (5.3) holds;
(iii) $\Gamma_{G} (\varphi )\ge 0$, when $P_{n}\cdot Q_{m}>0$ and (7.6) holds, and for all $s\in [\alpha ,\beta ]$ (7.8) and (7.9) hold.

The following two results are mean-value theorems of the Lagrange and Cauchy type, respectively, and they are obtained in an analogous way to the theorems of the same type in the previous sections, so we omit the proof.

#### Theorem 8.6

*Let* $[\alpha , \beta ]\subseteq \mathbb{R}$ *be an interval and* $c\in \langle \alpha ,\beta \rangle $. *Let* $x_{i}\in [a_{1},b_{1}] \subseteq [\alpha ,c]$, $p_{i}\in \mathbb{R}$ ($i=1,\ldots,n$) *be such that* $P_{n}\neq 0$ *and let* $y_{j}\in [a_{2},b_{2}]\subseteq [c, \beta ]$, $q_{j}\in \mathbb{R}$ ($j=1,\ldots,m$) *be such that* $Q_{m}\neq 0$. *Let* $\Gamma_{J_{D}}$, $\Gamma_{ELR_{D}}$, *and* $\Gamma_{G}$ *be the linear functionals defined above*, *and let* $\varphi \in C^{3}([\alpha ,\beta ])$.

(i) *If* (3.1) *holds and for all* $s_{1}\in [ \alpha ,c]$, $s_{2}\in [c,\beta ]$ (3.2) *holds*, *then there exists* $\xi_{1} \in [\alpha ,\beta ]$ *such that*
  8.10 $$\begin{aligned} \Gamma_{D} (\varphi )=\dfrac{\varphi '''(\xi_{1})}{6} \Biggl[\frac{1}{Q _{m}} \sum_{j=1}^{m}q_{j}y_{j}^{3}- \frac{1}{P_{n}}\sum_{i=1}^{n}p_{i}x _{i}^{3} +\bar{x}^{3}-\bar{y}^{3} \Biggr] . \end{aligned}$$
(ii) *If* (5.1) *holds*, *and for all* $s\in [ \alpha ,c]$ (5.2) *holds and for all* $s\in [c, \beta ]$ (5.3) *holds*, *then there exists* $\xi_{2} \in [\alpha ,\beta ]$ *such that*
  8.11 $$\begin{aligned} \Gamma_{ELR_{D}} (\varphi ) ={}&\dfrac{\varphi '''(\xi_{2})}{6} \Biggl[ \frac{b _{2}-\bar{y}}{b_{2}-a_{2}}a_{2}^{3}+\frac{\bar{y}-a_{2}}{b_{2}-a_{2}}b _{2}^{3}-\frac{1}{Q_{m}}\sum _{j=1}^{m} q_{j} y_{j}^{3} \\ & {}-\frac{b_{1}-\bar{f}}{b_{1}-a_{1}}a_{1}^{3}-\frac{\bar{f}-a_{1}}{b _{1}-a_{1}}b_{1}^{3}+ \frac{1}{P_{n}}\sum_{i=1}^{n} p_{i} x_{i}^{3} \Biggr]. \end{aligned}$$
(iii) *If* $P_{n}\cdot Q_{m}>0$ *and* (7.6) *holds*, *and for all* $s\in [\alpha ,\beta ]$ (7.8) *and* (7.9) *hold*, *then there exists* $\xi_{3} \in [\alpha , \beta ]$ *such that*
  8.12 $$\begin{aligned} \Gamma_{G} (\varphi ) ={}&\dfrac{\varphi '''(\xi_{3})}{6} \Biggl[A_{2} \Biggl( \sum_{j=1}^{m} q_{j}y_{j} \Biggr)^{3}+B_{2} \Biggl( \sum_{j=1}^{m} q _{j}-1 \Biggr) y_{0}^{3}-\sum _{j=1}^{m} q_{j}y_{j}^{3} \\ & {}- A_{1} \Biggl( \sum_{i=1}^{n} p_{i}x_{i} \Biggr)^{3}-B_{1} \Biggl( \sum_{i=1}^{n} p_{i}-1 \Biggr) x_{0}^{3}+\sum_{i=1}^{n} p_{i}x_{i}^{3} \Biggr]. \end{aligned}$$

#### Theorem 8.7

*Let the conditions of Theorem* 8.6 *hold and let* $\varphi , \psi \in C^{3}([\alpha ,\beta ])$. *If* $\Gamma_{J_{D}} ( \psi )\neq 0$, $\Gamma_{ELR_{D}} (\psi )\neq 0$, *and* $\Gamma_{G} ( \psi )\neq 0$, *then there exist* $\xi_{1},\xi_{2},\xi_{3} \in [\alpha ,\beta ]$ *such that all of the following statements hold*:

8.13 $$\begin{aligned}& \dfrac{\Gamma_{J_{D}} (\varphi )}{\Gamma_{J_{D}} (\psi )} =\dfrac{ \varphi '''(\xi_{1})}{\psi '''(\xi_{1})} \quad \textit{or}\quad \varphi '''( \xi_{1})=\psi '''( \xi_{1})=0, \end{aligned}$$

8.14 $$\begin{aligned}& \dfrac{\Gamma_{ELR_{D}} (\varphi )}{\Gamma_{ELR_{D}} (\psi )} =\dfrac{ \varphi '''(\xi_{2})}{\psi '''(\xi_{2})} \quad \textit{or}\quad \varphi '''( \xi_{2})=\psi '''( \xi_{2})=0, \end{aligned}$$

8.15 $$\begin{aligned}& \dfrac{\Gamma_{G} (\varphi )}{\Gamma_{G} (\psi )} =\dfrac{\varphi '''( \xi_{3})}{\psi '''(\xi_{3})} \quad \textit{or} \quad \varphi '''( \xi_{3})=\psi '''( \xi_{3})=0. \end{aligned}$$

#### Remark 8.8

Note that if in Theorem 8.7 we set the function *ψ* to be $\psi (x)=x^{3}$, we get exactly Theorem 8.6.

As a consequence of the previous two theorems, we now give some further results in which we give explicit conditions on $p_{i}, x_{i}$ ($i=1,\ldots,n$) and $q_{j}, y_{j}$ ($j=1,\ldots,m$) for (8.10) and (8.13) to hold, where using the properties of the function *G* we can skip the supplementary conditions on that function.

#### Corollary 8.9

*Let* $x_{i}\in [\alpha ,c]$, $p_{i}\in \mathbb{R}^{+}$ ($i=1,\ldots,n$) *and* $y_{j}\in [c,\beta ]$, $q_{j}\in \mathbb{R}^{+}$ ($j=1,\ldots,m$), *and let* $\varphi ,\psi \colon [\alpha ,\beta ]\to \mathbb{R}$.

(i) *If* (3.1) *holds and* $\varphi \in C^{3}([ \alpha ,\beta ])$, *then there exists* $\xi \in [\alpha ,\beta ]$ *such that* (8.10) *holds*.
(ii) *If* (3.1) *holds and* $\varphi ,\psi \in C ^{3}([\alpha ,\beta ])$, *then there exists* $\xi \in [\alpha ,\beta ]$ *such that* (8.13) *holds*.

#### Proof

Note that $p_{i},q_{j}>0$ implies that $\bar{x}\in [ \alpha ,c]$ and $\bar{y}\in [c,\beta ]$, so we can set the interval $[a_{1},b_{1}]$ to be $[\alpha ,c]$ and $[a_{2},b_{2}]$ to be $[c,\beta ]$. The function *G* is convex, so by Jensen’s inequality we see that the inequalities in (3.2) hold for all $s_{1} \in [\alpha ,c]$, $s_{2}\in [c,\beta ]$. Now we can apply Theorem 8.6 and Theorem 8.7 to get the statements of this corollary. □

#### Corollary 8.10

*Let* $(x_{1},\ldots,x_{n})$ *be monotonic* *n*-*tuple*, $x_{i}\in [ \alpha ,c]$ ($i=1,\ldots,n$) *and* $(y_{1},\ldots,y_{m})$ *be monotonic* *m*-*tuple*, $y_{j}\in [c,\beta ]$ ($j=1,\ldots,m$). *Let* $(p_{1},\ldots,p_{n})$ *be a real* *n*-*tuple such that*

$$ 0\leq P_{k} \leq P_{n} \quad (k=1,\ldots,n) , P_{n}>0, $$

*and* $(q_{1},\ldots,q_{m})$ *be a real* *m*-*tuple such that*

$$ 0\leq Q_{k} \leq Q_{m} \quad (k=1,\ldots,m) ,Q_{m}>0. $$

*Let* $\varphi ,\psi \colon [\alpha ,\beta ]\rightarrow \mathbb{R}$.

(i) *If* (3.1) *holds and* $\varphi \in C^{3}([ \alpha ,\beta ])$, *then there exists* $\xi \in [\alpha ,\beta ]$ *such that* (8.10) *holds*.
(ii) *If* (3.1) *holds and* $\varphi ,\psi \in C ^{3}([\alpha ,\beta ])$, *then there exists* $\xi \in [\alpha ,\beta ]$ *such that* (8.13) *holds*.

#### Proof

Suppose that $x_{1}\geq x_{2}\geq \cdots \geq x_{n}$. We have

$$\begin{aligned} P_{n}(x_{1}-\bar{x})=\sum_{i=2}^{n} p_{i} (x_{1}-x_{i})=\sum _{j=2} ^{n} (x_{j-1}-x_{j}) (P_{n}-P_{j-1})\geq 0 \end{aligned}$$

so it follows that $x_{1}\geq \bar{x}$. Furthermore,

$$\begin{aligned} P_{n}(\bar{x}-x_{n})=\sum_{i=1}^{n-1} p_{i} (x_{i}-x_{n})=\sum _{j=1} ^{n-1} (x_{j}-x_{j+1})P_{j} \geq 0, \end{aligned}$$

so $\bar{x}\geq x_{n}$. We see that we have obtained $x_{n}\le \bar{x}\le x_{1}$, that is, $\bar{x}\in [\alpha ,c]$. In an analogous way we can get $\bar{y}\in [c,\beta ]$. Therefore, as well as in the proof of the previous corollary, we can set the interval $[a_{1},b _{1}]$ to be $[\alpha ,c]$ and $[a_{2},b_{2}]$ to be $[c,\beta ]$. By the Jensen-Steffensen inequality we see that for the convex function *G* the inequalities in (3.2) hold for all $s_{1}\in [ \alpha ,c]$, $s_{2}\in [c,\beta ]$. Now the statements of this corollary follow directly from Theorem 8.6 and Theorem 8.7. □
